# Winter cover crops increased nitrogen availability and efficient use during eight years of intensive organic vegetable production

**DOI:** 10.1371/journal.pone.0267757

**Published:** 2022-04-28

**Authors:** Kathryn E. White, Eric B. Brennan, Michel A. Cavigelli, Richard F. Smith

**Affiliations:** 1 United States Department of Agriculture, Agricultural Research Service, Sustainable Agricultural Systems Laboratory, Beltsville Agricultural Research Center, Beltsville, Maryland, United States of America; 2 United States Department of Agriculture, Agricultural Research Service, Salinas, California, United States of America; 3 University of California Cooperative Extension, Salinas, California, United States of America; Government College University Faisalabad, Pakistan, PAKISTAN

## Abstract

Efficient use of nitrogen (N) is essential to protect water quality in high-input organic vegetable production systems, but little is known about the long-term effects of organic management on N mass balances. We measured soil N and tabulated N inputs (organic fertilizers, compost, irrigation water, atmospheric deposition, cover crop seed, vegetable transplant plugs and fixation by legume cover crops) and exports in harvested crops (lettuce, broccoli) over eight years to calculate soil surface and soil system N mass balances for the Salinas Organic Cropping Systems study in Salinas, CA. Our objectives were to 1) quantify the long-term effects of compost, cover crop frequency and cover crop type on soil N, cover crop and vegetable crop N uptake, and yield, and 2) tabulate N balances to assess the effects of these factors on N export in harvested crops, soil N storage and potential N loss. Results show that across all systems only 13 to 23% of N inputs were exported in harvest. Annual compost applications increased soil N stocks but had little effect on vegetable N uptake or yield, increasing the cumulative soil system N balance surplus over eight years by 999 kg ha^-1^, relative to the system receiving organic fertilizers alone. Annually planted winter cover crops increased N availability, crop uptake and export; however, biological N fixation by legumes negated the positive effect of increased harvest exports on the balance surplus in the legume-rye cover cropped system. Over eight years, rye cover crops improved system performance and reduced the cumulative N surplus by 384 kg ha^-1^ relative to the legume-rye mixture by increasing N retention and availability without increasing N inputs. Reduced reliance on external compost inputs and increased use of annually planted non-legume cover crops can improve efficient N use and cropping system yield, consequently improving environmental performance.

## Introduction

Efficient use of nitrogen (N) in agricultural systems is essential for optimizing yield, economic performance and environmental sustainability. Nutrient mass balances, including soil surface and soil system balances, are important tools to assess nutrient use efficiency and potential loss pathways by examining N flows within and across defined system boundaries [1–5]. For example, a recent meta-analysis found that N balance surplus was a better predictor of soil N_2_O emissions compared to N application rate in vegetable and grain crops [6]. Soil surface balances, which account for nutrient inputs to the soil and crop nutrient exports, provide insights about input use efficiency in harvested crops and potential soil nutrient extraction when balances are negative or about soil nutrient loading when balances are positive. Soil system balances, which also account for soil nutrient stocks, internal cycling, and losses, integrate nutrient inputs and outputs with nutrient recycling in crop and cover crop residues and soil storage or loss [3]. For example, balance surpluses in sandy soils may indicate increased risk of N leaching losses [7] due to limited organic matter storage [8] and low nutrient retention capacity [9] coupled with rapid water percolation due to limited water holding capacity and high permeability [10].

Production of multiple crops per year in intensive organic vegetable systems such as those in the Salinas Valley, CA, USA currently use large nutrient applications including compost, pelleted organic fertilizers and liquid organic fertilizers [11–13]. These products differ in N availability, some releasing N relatively rapidly, and others increasing soil N storage, potentially mineralizable N and N availability for crop uptake over time [14–18]. However, these frequent large inputs, which are often considered necessary to ensure sufficient N availability [19, 20] for these the high economic value vegetable crops, can result in positive soil surface N balances and unsustainable soil N loading [12]. As a result, N mineralized asynchronously with or in excess of crop uptake may be eventually lost from the system [12]. Excess mineral N losses from decades of inefficient fertilizer use in intensive high input vegetable production systems in California—largely from conventional systems—have resulted in groundwater contamination, impacting both irrigation and drinking water quality [21]. Winter cover crops are an effective management strategy to reduce N losses in intensive Salinas production systems [22, 23]. Farmers in California’s central coast region are under increasing scrutiny to reduce N fertilizer inputs, and cover crops will likely play a major role in this effort [24]. The impact of current organic management strategies on N balances in intensive high input organic systems in California has not been well documented [21]. This is an important issue because organic production accounted for 13% of the $ U.S. 4.4 billion of agricultural production in Monterey county, which includes the Salinas Valley, in 2019 [25]. Due to the differential N availability from organic fertilizers, evaluating the risk of N loss under organic management requires a long-term approach that assesses the complex balance of N inputs, exports, internal cycling and storage, availability and export over multiple growing seasons [2, 5].

A fundamental aim of organic agriculture is to build long-term soil fertility and rely on ecological processes to maintain soil N availability [14, 26]. The goal is to maintain crop yield by fostering efficient cycling of N between labile and slowly available soil organic matter (SOM) pools and microbial biomass to provide plant available N in synchrony with crop demand while minimizing N losses through volatilization, leaching, denitrification, surface runoff and erosion [5, 12, 27, 28]. However, achieving this goal in high input organic vegetable systems can be difficult. For example, on farms that relied on amendments such as manure and compost rather than more readily available organic fertilizers (feather and seed meals, sugar processing wastes, etc.) total N applications were 88% greater, resulting in a 335% greater positive N balance in German organic production [11].

Cover crops can help increase nutrient use efficiency by retaining excess mineral N after cash crop harvest and increasing N availability to the subsequent cash crops [29–33]. For example, nonlegume cover crops following broccoli in Salinas Valley production systems decreased winter nitrate N (NO_3_-N) leaching by 65–70% [23]. In contrast, winter N leaching losses were high in a modelling study with several organically managed broccoli systems (111 to 343 kg N ha^-1^; depending on organic fertilizer and compost input) and was not reduced by legume-cereal cover crops [34]. Though non-legume cover crops have the greatest capacity to reduce leaching losses [22, 29, 33, 35], N mineralization and availability following incorporation is influenced by the C:N ratio of cover crop species [32, 36]. Legume cover crops fix atmospheric N, increasing both labile soil N and crop N uptake after termination [17, 37–41], but their ability to scavenge residual N is limited [29]. Mixtures of legumes and non-legumes can provide the benefits of both functional groups, but effects on N leaching losses and N availability are variable [42–45] and depend on the biomass ratio of legumes to non-legumes and the C:N ratio of that biomass [46]. Long-term cover crop biomass incorporation increases fast cycling labile soil organic carbon (SOC) and organic N [17, 47–49], thereby increasing soil N availability as SOM mineralizes [5, 50]. Cover crops and cover crop mixtures also increase the size of the soil microbial biomass pool, microbial activity, and microbial biomass N [51–53]. Microbial processing of cover crop residues can also increase the size of the slowly cycling SOC pool, increase soil N storage and availability [5, 48], and reduce the need for additional external inputs.

The individual and combined effects of cover cropping and compost application on soil N and crop N uptake and export in organic vegetable systems remains largely unexplored. For example, how do relatively large N inputs from fertilizers and compost, combined with intensive tillage common in organic cool season vegetable systems, interact with cover crop effects? The Salinas Valley, where this study occurred, is one of the most important regions for cool season vegetable production in the United States. For example, California is the top lettuce and broccoli production area in the U.S., and most of this occurs in the Salinas Valley [54]. Frequent intensive tillage operations used by Salinas Valley vegetable producers accelerate SOC mineralization and associated N mineralization, thereby increasing the potential for N loss [35, 47]. Mass balances for long-term studies such as the Salinas Organic Cropping System (SOCS) experiment–the focus of this paper–provide insights on the interaction of fertility amendments, SOM and cropping system management over time. This information is essential to develop improved management practices that promote efficient N use and maintain crop yield while minimizing surplus N losses to the environment.

The objectives of this paper are to 1) quantify the long-term effects of compost, cover crop frequency and cover crop type on soil N, cover crop N uptake, crop N uptake and yield during 8 years of intensive organic vegetable production in the Salinas Valley, CA and 2) tabulate N balances to assess the effects of these treatment factors on N export in harvested crops, soil N storage and potential N loss to improve N management in organic vegetable systems and protect water quality.

## Materials and methods

### Ethics statement

This study was part of a USDA-ARS internally funded research project conducted at a USDA-ARS research facility therefore no special permissions were required to conduct the research at this site. The field studies did not involve endangered or protected species.

### Field site and management

The field site, its history and the SOCS experimental design are described in detail by Brennan and Boyd [33]. The study was initiated in 2003 on a Chualar loamy sand (fine-loamy, mixed, superactive, thermic Typic Argixerol; 77% sand, 15% silt, 8% clay) at the USDA-ARS in Salinas, CA, USA (36°37ʹN, -121°32ʹW). The site has been certified organic since 1999 by California Certified Organic Farmers and managed under the requirements of the USDA National Organic Program since 2002. In the year prior to establishing the study, the field was winter cover cropped with a legume-rye mixture [10% ‘Merced’ rye (*Secale cereale* L.), 35% faba bean (*Vicia faba* L.), 25% ‘Magnus’ pea (*Pisum sativum* L.), 15% common vetch (*Vicia sativa* L.) and 15% purple vetch (*Vicia benghalensis* L.)] and then summer cover cropped with vetch-mustard (95% common vetch, 5% *Brassica juncea* Czern.) and then buckwheat (*Fagopyrum esculentum* Moench); these cover crops were mowed and disked into the soil. In addition, in the year prior to the study the soil was amended with approximately 22 Mg ha^-1^ (wet weight) urban yard waste compost and approximately 12.3 Mg ha^-1^ mined Lima gypsum that were broadcast and incorporated; gypsum was added to improve soil structure, reduce crusting and increase water infiltration. We report compost wet weight because the moisture content of the material applied was not measured. The C:N ratio of the compost applied before the experiment began was not determined but was likely within the range (18–24) of the similar compost used during the experiment.

The experimental design is a randomized complete block with eight cropping systems that differ by cover crop type, cover crop seeding rate, and compost application in 19.5 m x 12.2 m plots arranged in four blocks. Management of all systems reflects the practices used by regional commercial growers. The five systems in Table 1 are the same systems previously examined for soil carbon, phosphorus, microbial community dynamics and soil enzyme activity [47, 51, 52, 55]. Systems 1 and 2 were cover cropped with legume-rye during the winter prior to years 4 and 8 and were left fallow all other winters; weeds that grew during the winter fallow were controlled by hand weeding and shallow tillage as needed. Systems 3, 4 and 5 were cover cropped each winter (October or November to February or March) [56]. Cover crops were planted at 15 cm spacing using a grain drill equipped with cones to facilitate planting the various cover crop types at different rates [57].

**Table 1:** The five systems at the Salinas Organic Cropping Systems (SOCS) experiment evaluated in this analysis.

System	Compost Rate^1^	Cover Crop Type	Cover Crop Frequency
	Mg ha^-1^ vegetable crop^-1^		
1	--	Legume-rye^2^	Quadrennial
2	7.6	Legume-rye^2^	Quadrennial
3	7.6	Legume-rye^2^	Annual
4	7.6	Mustard^3^	Annual
5	7.6	Rye^4^	Annual

^1^ Oven-dry cumulative annual compost application rate: 15.2 Mg ha^-1^ during years 1–7 when two vegetable crops were grown and half this during year 8 when only one vegetable crop was grown.

^2^ Seeding rate: 420 kg ha^-1^, 10% ‘Merced’ rye, 35% faba bean, 25% ‘Magnus’ pea, 15% common vetch and 15% purple vetch by seed weight.

^3^ Seeding rate: 11 kg ha^-1^; 61% ‘Ida Gold’ white mustard (*Sinapis alba* L.), 39% ‘Pacific Gold’ Indian mustard (*Brassica juncea* Czern.) by seed weight.

^4^ Seeding rate: 90 kg ha^-1^; ‘Merced’ rye alone.

All plots, regardless of cover crop treatment, were tilled with a soil spader in February to March of each year to incorporate cover crops (when present) and prepare the soil for bed formation. Cover crops were terminated after flowering of rye, mustard, and most legumes had begun. Following tillage, cover crops were left to decompose for 30 to 42 days, except for year 6 when the period was 72 days due to wet spring weather. Following this period, lister plows were used to form 101.6 cm wide peaked beds. Systems 2, 3, 4 and 5 received 7.6 Mg ha^-1^ (oven-dry weight basis) urban yard waste compost (Z-Best Products, Gilroy, CA), while all systems received pelleted poultry manure/feather meal based organic fertilizer (Foster Poultry Farms 4-4-2, Livingston, CA; True Organic 8-1-1, Helm, CA) to provide 55–66 kg N ha^-1^; the pelleted fertilizer was injected into two lines in the peaked beds. The compost, which was analyzed in 4 of the 8 years, had total N and P concentrations of 15 g kg^-1^ N and 2.5 g kg^-1^ P, and a C:N ratio ranging from 18 to 28 with a mean of 22. The compost was made from urban yard waste from the region where the study occurred. Beds were shaped to incorporate the compost, creating approximately 50 cm wide flat bed tops. Transplanted romaine lettuce (*Lactuca sativa* L. var. *longifolia* Lam.) was grown from May to June each year and fertigated with liquid organic fertilizer (Biolizer GP 2.5-2-1.5, California Liquid Fertilizer, Gonzales, CA; Agrolizer 6-2-0, AgroMar, San Diego, CA; Tierra Fertil 5-1-1, Mar Y Tierra Fertilizantes Orgánicos, Ensenada, Mexico). Annual application of N to lettuce in pelleted and liquid fertilizers was 73 kg ha^-1^. After the lettuce harvest, beds were either reshaped or flattened and reformed depending on field conditions and an additional 7.6 Mg ha^-1^ of yard waste compost was incorporated in Systems 2, 3, 4 and 5 to prepare for the second summer crop [58]. Baby leaf spinach was planted after lettuce in year 1 (July–August 2004) while transplanted broccoli was grown in all other years (July–September/October) except in year 8 when the field was transitioned to strawberry production. Spinach received 22 kg N ha^-1^ as pelleted organic fertilizer, while broccoli received pelleted and liquid fertilizers totaling 134 to 170 kg N ha^-1^ y^-1^. Crops were harvested and marketed by collaborating farms, except for the 2004 spinach and 2005 broccoli crops, which did not meet market standards. Lettuce crops were harvested in June to July, and broccoli was harvested in September to October. These crops and all harvest residues in other years were incorporated into the soil by disking, spring-tooth harrowing, soil spading and ring rolling prior to the winter fallow or cover crop treatments. The planting dates for all cover crops and vegetables, as well as the dates for cover crop termination and vegetable harvest were described by Brennan and Boyd [33].

### Nitrogen inputs

Cumulative N input from fertilizers, compost, transplant potting mix and cover crop seed was calculated from the measured cumulative mass of each material applied and its N concentration (Table 2). Total N concentrations for the pelleted and liquid fertilizers were taken from the manufacturers’ analyses. Concentrations in all other inputs were determined by dry combustion analysis of dried (105°C) and ground (0.250 mm) samples by the University of California-Davis Analytical Laboratory following additional sample drying at 105°C (http://anlab.ucdavis.edu/analyses/plant/522) with a TruSpec CN analyzer (LECO Corp., Saint Joseph, MI). Cumulative N from atmospheric deposition was obtained from the nearest National Atmospheric Deposition Program monitoring station at Pinnacles National Monument (Site CA-66), which is 37 km from the study site. Legume N fixation in Systems 1, 2 and 3 was estimated from the sum of legume shoot N uptake and estimated root N uptake (see below) multiplied by the fraction of legume N derived from the atmosphere, obtained from the literature [59]. Nitrogen in irrigation water was determined based on the NO_3_-N concentration of the well water (4 ppm) and irrigation application rates for each crop as follows (mm ha^-1^): cover crop (34), lettuce (181), spinach (152), broccoli (335). Irrigation rates were measured for spinach in year 1 and for lettuce, broccoli and cover crops from years 3 to 8. Average irrigation rates for lettuce and broccoli in years 3 to 8 were assumed for years 1 and 2. Application rates were uniform across all crops in all systems including during the winter fallow periods when Systems 1 and 2 received the same amount of irrigation water as systems with annual cover crops. Irrigation rates for a given crop included water applied during growth of crops and prior to field work such as bed shaping. Irrigation rates during vegetable production were based on daily monitoring of soil moisture using soil moisture sensors installed at 20 and 46 cm depths and evapotranspiration (ET) data from a nearby weather station of the California Irrigation Management Information System.

**Table 2:** Total nitrogen concentrations of inputs at the Salinas Organic Cropping Systems (SOCS) experiment.

Input Source	Total Nitrogen
	g kg^-1^
4-4-2 Pelleted Fertilizer	40.0
8-1-1 Pelleted Fertilizer	80.0
2.5-2-1.5 Liquid Fertilizer (Years 2 to 3)	25.0
6-2-0 Liquid Fertilizer (Years 4 to 5)	60.0
5-1-1 Liquid Fertilizer (Years 6 to 8)	50.0
Compost	15.0
Transplant Potting Mix	4.9
*Cover Crop Seed*	
‘Merced’ Winter Rye	28.9
‘Pacific Gold’ Mustard	41.6
‘Ida Gold’ Mustard	53.5
‘Magnus’ Pea	35.8
Purple Vetch	49.8
Common Vetch	46.7
Faba Bean	45.2

### Nitrogen uptake

Total cover crop soil N uptake was determined using previously published cover crop shoot biomass production and N concentration data [33]. Cover crop biomass was sampled from one 50 by 100 cm quadrat oriented to include three adjacent rows from each plot prior to cover crop incorporation. The legume-rye cover crop samples were separated into legume and rye components. Biomass N contents were determined by multiplying cumulative cover crop shoot and estimated root dry matter by its N concentration determined by dry combustion analysis of dried (105°C) and ground (0.250 mm) samples by the University of California-Davis Analytical Laboratory as described above for N inputs. Below ground biomass was estimated based on above ground biomass and shoot:root ratios from the literature for the cover crops. We assumed shoot:root ratios of 5.6 for rye [22, 23], 4.5 for legumes [60] and 6.3 for mustard [22]. Legume-rye root biomass is the sum of the root biomass of each component. We assumed that below ground biomass had 20% lower N concentration than that measured in the above ground biomass [61]. To separately assess soil N uptake by the legume-rye cover crop and N fixation as a new N input, estimated legume N derived from the atmosphere by N fixation was subtracted from legume-rye N uptake. Estimated inputs from biological N fixation are included as an input in the N balances, whereas soil N captured by cover crops and reincorporated into soil organic matter is accounted by changes to soil N stocks as described below.

The vegetable post-harvest residues were estimated based on mature lettuce and broccoli oven-dry shoot biomass assuming harvest indices of 0.26 and 0.24 for romaine lettuce hearts and broccoli, respectively (Brennan and Smith, unpublished data). To estimate the N exported from the field in the harvested vegetables we multiplied the total shoot N content by the harvest index for lettuce, whereas for broccoli the total shoot N content was multiplied by 0.31 based on Smith et al. [20]. Lettuce and broccoli biomass were calculated based on 32 and 20 plants, respectively, harvested from each plot. Vegetable shoot N uptake was calculated from the biomass N concentrations in the above ground tissue measured for both lettuce and broccoli in each system every year by combustion analysis by the methods described above. We estimated below ground N uptake by vegetable roots based on above ground biomass and shoot:root ratios of the vegetables grown under similar field conditions in the Salinas Valley reported in the literature [22, 23, 62], and assuming that the N concentrations of the below ground biomass were 20% lower than in the above ground biomass. We assumed shoot:root ratios of 4.1 for lettuce [62] and 4.7 for broccoli [22, 23]. Vegetable residue N was calculated as the sum of belowground biomass N and the unharvested portion of above ground biomass N.

### Soil sampling and nitrogen analysis

Composite soil samples (0 to 30 cm depth) of 20 subsamples were collected each fall from each plot prior to cover crop planting or winter fallow. Total soil N (TN) was determined on air-dried ground (<0.5 mm) samples by combustion analysis as described above and nitrate-N (NO_3_-N) was determined using flow injection photometric analysis of 2.0 N KCl extracts (https://anlab.ucdavis.edu/analysis/Soils/312) using a Lachat QuikChem autoanayzer (Lachat Instruments, Loveland, CO). Soil TN stocks were calculated using the maximum equivalent soil mass method [63], which used the greatest bulk density measured during the study as a means of correcting for errors in stock measurements to a fixed depth due to changes in soil bulk density over time. This method allows comparisons based on the same soil mass per unit land area. Soil bulk density was measured between two broccoli plants in intact 5 cm by 12.8 cm samples in four separate beds per plot at the end of years 3 and 7 in each system (Brennan, unpublished data). A correction factor was applied to all bulk density measurements because the initial measurements were not corrected for the presence of small rocks and archived samples were not available. This correction factor was determined using samples collected in 2020. Samples were weighed, passed through a 2 mm sieve, and rock fragments retained on the sieve were weighed to determine the percentage of rock in each sample. The proportion of rock to soil was consistent among all samples. The volume of the rocks was measured by water displacement. We determined the relationship between rock mass and volume and used the result to determine rock density. Corrected bulk density was calculated as [64]:

$$Corrected\;Bulk\;Density=\frac{{Mass}_{core}-{Mass}_{rock}}{{Volume}_{core}-{Volume}_{rock}}$$

Given the intensity of tillage by soil spading to 30 cm, calculations were made with the assumption that bulk density was uniform throughout the 0 to 30 cm depth.

### Nitrogen balance

The soil surface N mass balance for each system over 8 years was calculated as the sum of cumulative total N inputs (from atmospheric deposition, cover crop seed, compost, liquid and pelleted fertilizers, transplant mix, legume N fixation, and irrigation water) minus the sum of cumulative N exports in harvested lettuce and broccoli. The soil system balance also accounts for changes in soil TN, which were calculated by subtracting the average System 1 soil TN stock (which received no compost) from the average TN stock of each of the other systems. The soil system balance was calculated as the difference between the soil surface balance and the change in soil N storage. This balance accounts for soil N storage only at the 0 to 30 cm depth and does not account for deeper soil storage or potential losses due to N leaching and denitrification.

### Statistical analyses

Statistical analyses were conducted using SAS ver. 9.4 (SAS Inst. Cary, NC) and the Exploratory Software for Confidence Intervals (ESCI) [65]. The raw data along with means and 95% confidence intervals (CI) for the 5 systems are presented to better visualize variability, scatter and skewness as suggested by Drummond and Vowler [66]. To avoid the over-reliance on null hypothesis statistical testing documented in the literature [67–74], we evaluated treatment effects using the CIs of the paired differences between systems [67, 68]. This allowed us to evaluate the effect size of the experimental factors, compost, cover crop frequency and cover crop type. For example, the CI of the mean difference between Systems 1 and 2 (no compost vs. annual compost) was used to evaluate the compost effect, and the CI of the difference between Systems 2 and 3 (quadrennial vs. annual legume-rye) was used to test the effect of cover cropping frequency. We calculated a standardized effect size measure (Cohen’s unbiased *d*, hereafter “*d*_unb_”) by dividing the effect size in original units by a standardizer that is based on the standard deviations of the two paired measurements and multiplied by an adjustment factor in ESCI [67]. This standardized effect size can be used to compare effects regardless of differences in the scale of the units of measurement.

When comparing treatment effects using this approach, a CI of a paired difference that does not include zero likely indicates a true effect (i.e. P<0.05 comparison-wise error rate) [67, 68]. If a CI of a paired difference just touches zero, then P = 0.05. While the relationship between CI and P-values serves as a point of reference, CIs should not be interpreted in a rigid, dichotomous way. Rather, the CIs should be viewed along with the distribution of the experimental data to evaluate a potential effect [68]. Brennan and Acosta-Martinez [52] provide a more detailed discussion of this inference approach, which is consistent with the data presentation used to evaluate SOCS treatment effects on soil total and labile soil C [47], MBC, MBN [51], and enzyme activity [52], and on cover crop N uptake, residue quality, and N mineralization [32, 33].

We also present means comparisons to provide readers with additional tools to evaluate results. Treatment effects on TN stock and NO_3_-N concentration over years 1 to 7 were assessed using a repeated measures ANOVA by the MIXED procedure fitted with a first order autoregressive covariance structure using SAS, with system, year, and system x year as fixed effects and block as a random effect. Cumulative N uptake by cover crop shoots, vegetable shoots and total N inputs over 8 years was analyzed by ANOVA, with system as a fixed effect and block as a random effect. Variances were grouped due to variance heterogeneity among the systems. Cumulative lettuce and broccoli yield, N export, soil N storage and both N balances were analyzed by ANOVA with system as a fixed effect and block as a random effect. Means separations for all ANOVAs with a significant F test were performed by the Tukey-Kramer test (P≤0.05), which controls for family-wise error.

## Results

### Change in soil total nitrogen stocks

**Fig 1:**
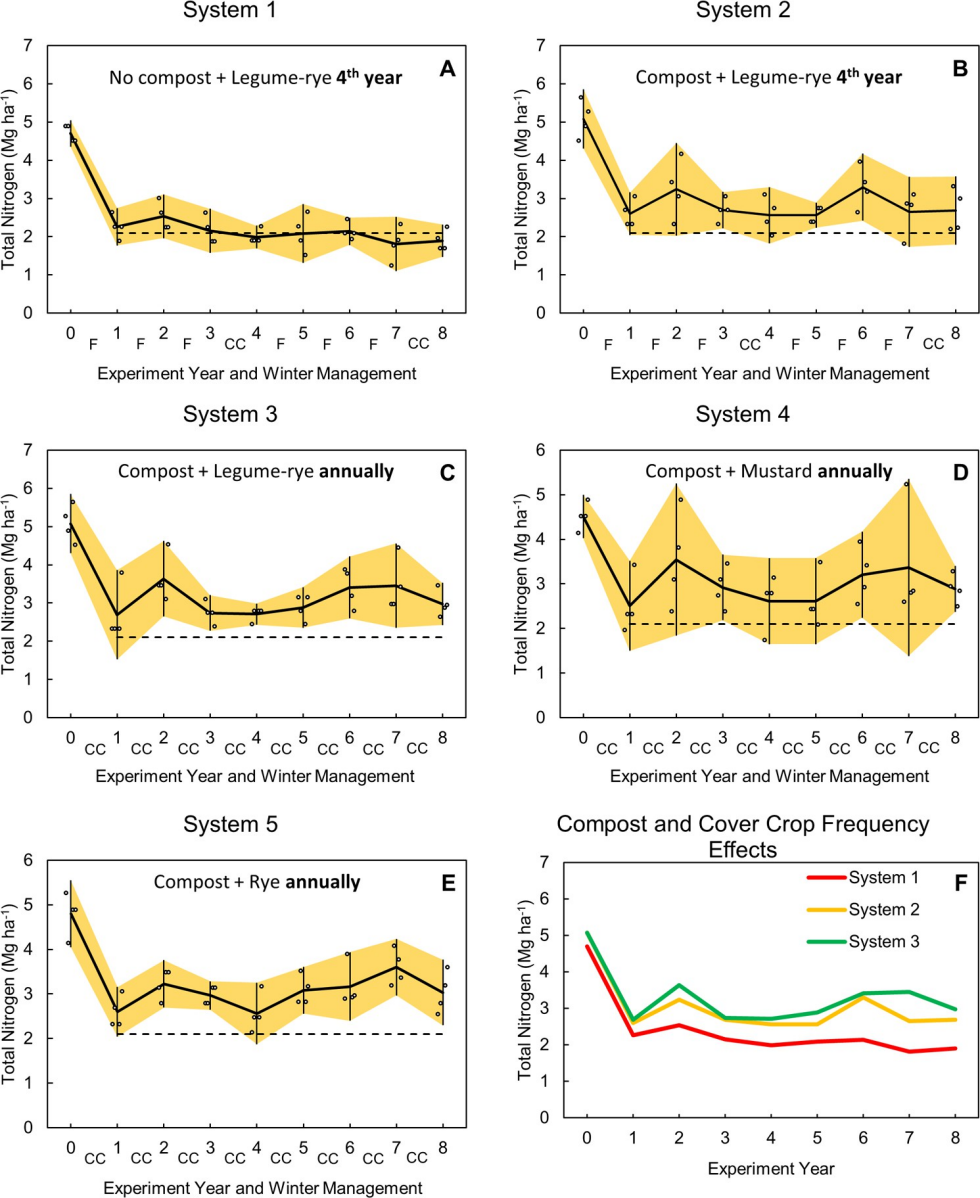
Soil total nitrogen stocks at the 0 to 30 cm depth by system and year. Systems differed by annual compost additions, cover crop type and frequency. The horizontal dashed line at 2.1 Mg ha^-1^ is the mean soil total nitrogen between years 1 and 8 for System 1 and is included on each graph as a reference. Error bars are 95% confidence intervals (CI) that are connected from year to year by the yellow band. Individual data points for replicates 1 through 4 of each system are clustered around the mean in order from left to right so as not to obscure the CI. The F and CC labels along the x-axis indicate when the system was fallow (F) or cover cropped (CC). The soil was sampled in the fall each year right before the winter cover cropping or fallow period (beginning at time 0); therefore, the 4^th^ and 8^th^ sampling dates for Systems 1 and 2 occurred right before they received their first and second cover crops.

**Fig 2:**
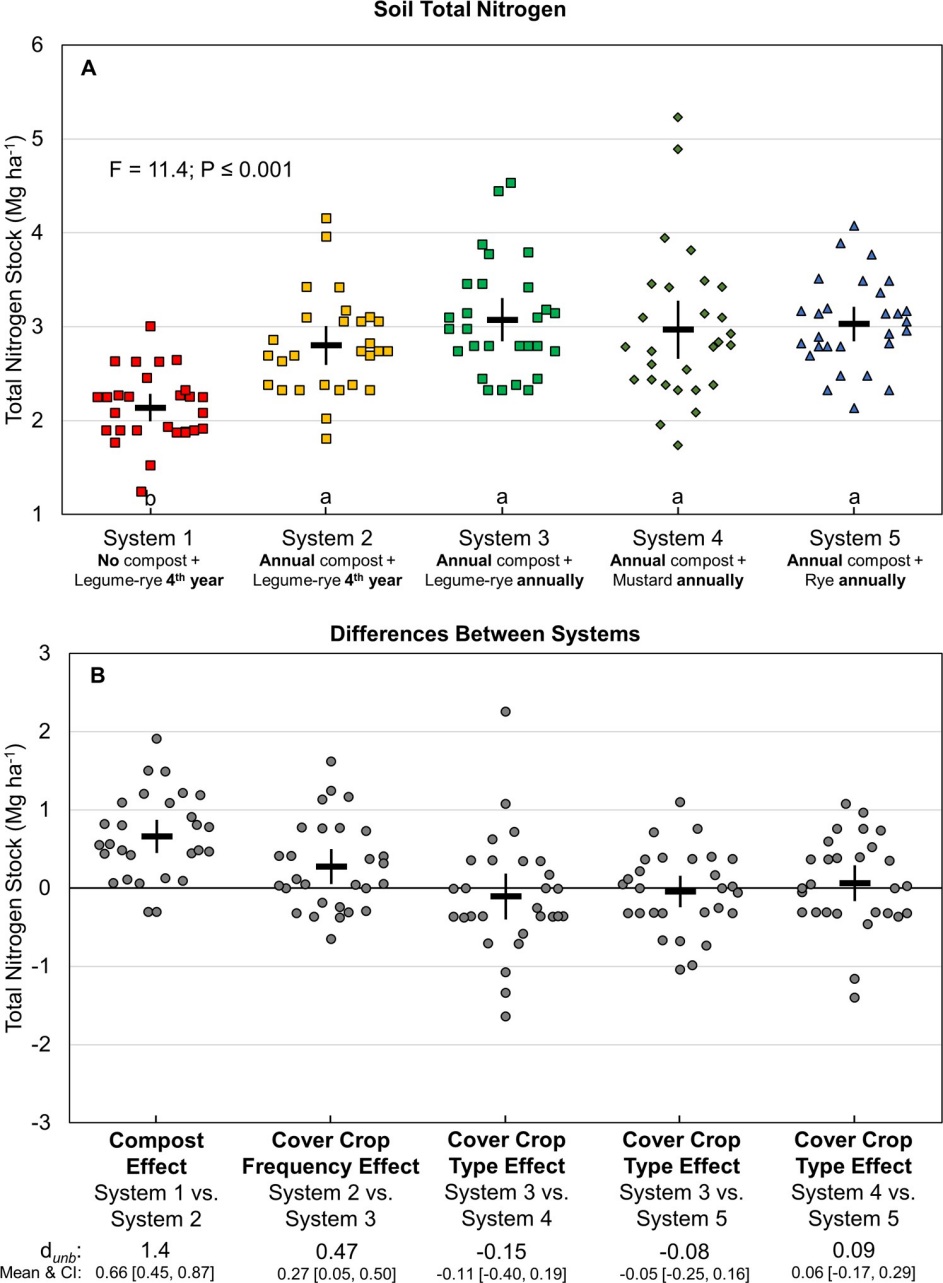
Soil total nitrogen stocks over seven years of vegetable production. Mean soil total nitrogen stocks (A) and mean paired differences between systems (B). Systems differed by annual compost additions, cover crop type and cover cropping frequency. Error bars are 95% confidence intervals (CI) around the mean (horizontal line). Samples were taken at the 0 to 30 cm depth after the 2^nd^ vegetable crop before winter fallow or cover cropping for years 1 to 7 when two vegetable crops were grown annually. Data points are the 4 replicates each year and are clustered around the mean so as not to obscure the mean and CI. Different lower-case letters above the x-axis indicate that means are significantly different based on the Tukey-Kramer adjusted family-wise error rate of P≤0.05. The standardized effect size (Cohen’s unbiased *d*, *d*_*unb*_) is shown below the x-axis in plot B.

At the beginning of the study the mean soil N stock was 4.8 Mg ha^-1^ in the top 30 cm (Fig 1). In year 1 stocks declined, on average, by 48% to 2.5 Mg ha^-1^ across all systems, but the largest loss was in System 1, which did not receive compost. The horizontal dashed reference line in Fig 1 indicates the mean total soil N between years 1 and 8 for System 1 and is included as a helpful guide to compare systems. The positive effects of compost and cover crops on N stocks are illustrated by higher average N stocks in Systems 2 and 3 than in System 1 during years 1 to 8 (Fig 1F). These effects are also illustrated by the differences in soil N stocks among systems during years 1 to 7 (Fig 2). For example, among the legume-rye systems, compost had a nearly 3 times greater effect than increased cover crop frequency on mean soil N stocks (*d*_unb_ = 1.4 vs. 0.47; Fig 2B). In the annually cover cropped systems that received compost—Systems 3, 4 and 5—there was no clear evidence that cover crop type had an effect on N stocks.

### Residual soil nitrate nitrogen

**Fig 3:**
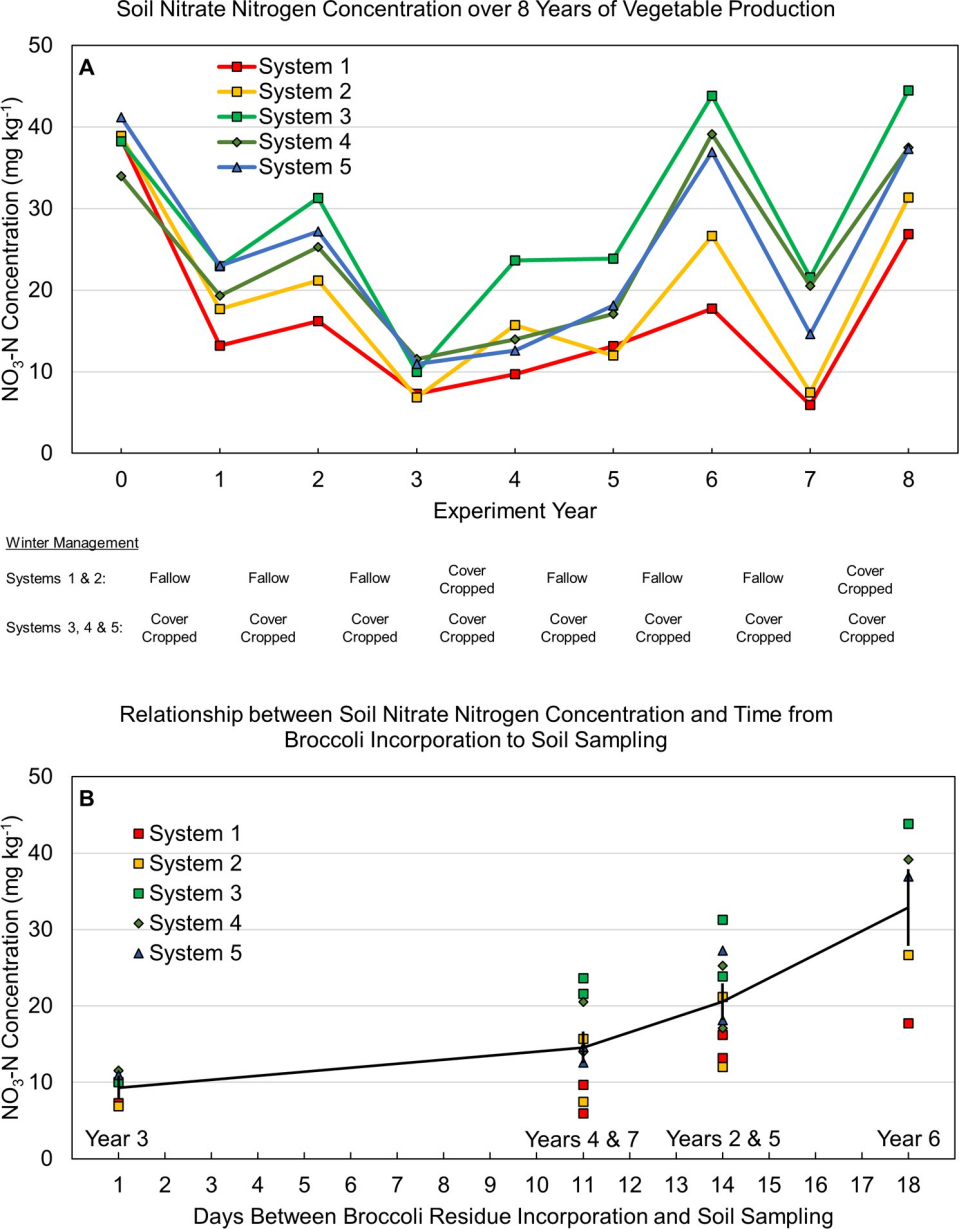
Post-harvest soil nitrate nitrogen concentration over 8 years of vegetable production. Samples were taken after incorporating the last vegetable crop each year and before cover cropping or fallow. Systems differed by annual compost additions, cover crop type and cover cropping frequency. Samples were taken at the 0 to 30 cm depth after the 2^nd^ vegetable crop before winter fallow or cover cropping for years 1 to 7 when two vegetable crops were grown annually (A). When broccoli was grown after lettuce (Years 2 to 7), the relationship between nitrate nitrogen concentrations and days after broccoli incorporation is shown (B); the individual data points represent the mean value for each system and the year(s) that the data correspond to is shown above the x axis. The error bars in plot B are 95% confidence intervals for the mean values.

**Fig 4:**
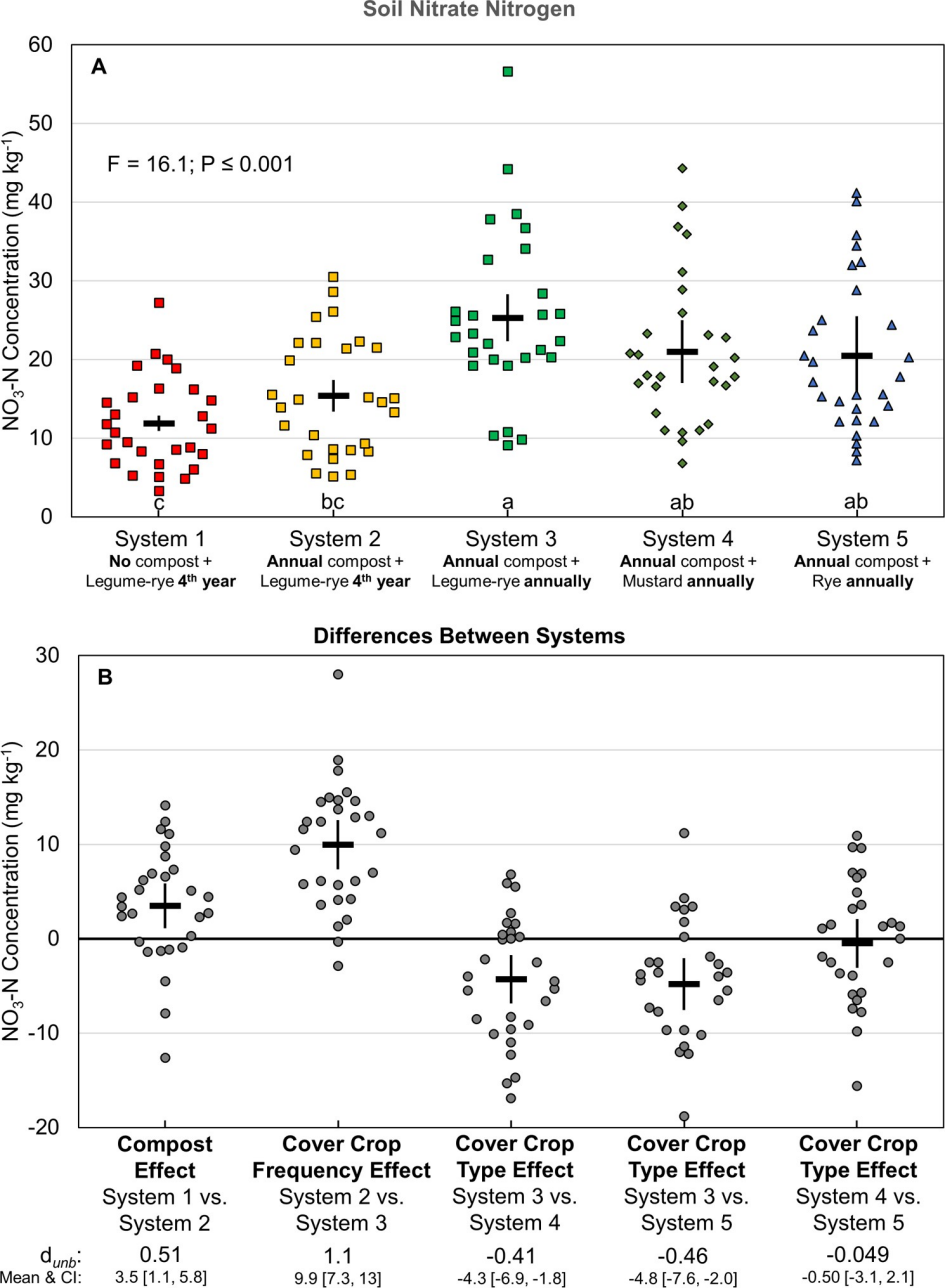
Post-harvest soil nitrate concentrations over seven years of vegetable production. Mean soil nitrate concentrations (A) and mean paired differences between systems (B). Samples were taken at the 0 to 30 cm depth after the 2^nd^ vegetable crop before winter fallow or cover cropping for years 1 to 7 when two vegetable crops were grown annually. Systems differed by annual compost additions, cover crop type and cover cropping frequency. Error bars are 95% confidence intervals (CI) around the mean (horizontal line). Data points are the 4 replicates each year and are clustered around the mean so as not to obscure the mean and CI. Different lower-case letters above the x-axis in plot A indicate that means are significantly different based on the Tukey-Kramer adjusted family-wise error rate of P≤0.05. The standardized effect size (Cohen’s unbiased *d*, *d*_*unb*_) is shown below the x-axis in plot B.

Soil NO_3_-N concentration prior to winter cover cropping or fallow—an indicator of NO_3_ leaching potential—varied considerably by year but was generally between 10 and 40 mg kg^-1^ (Fig 3A). The relatively large year-to-year variability followed similar patterns among systems (Fig 3B), suggesting that fluctuations were driven in part by factors other than treatment effects, such as weather patterns or differences in the timing of vegetable crop residue incorporation relative to soil sampling among years. In contrast to soil N stocks, annual legume-rye cover crops in System 3 increased post-harvest soil NO_3_-N more than did annual compost applications in System 2 (Fig 4), as indicated by *d*_unb_ = 1.1 vs. 0.51, respectively (Fig 4B). Residual soil NO_3_-N averaged over years 1 to 8 was 64% higher in System 3 than System 2. In addition, the legume-rye cover crop resulted in soil NO_3_-N that was 4 to 5 mg kg^-1^ greater than in systems with mustard (System 4) or rye (System 5) cover crops (Fig 4B).

### Nitrogen uptake by cover and cash crops

**Fig 5:**
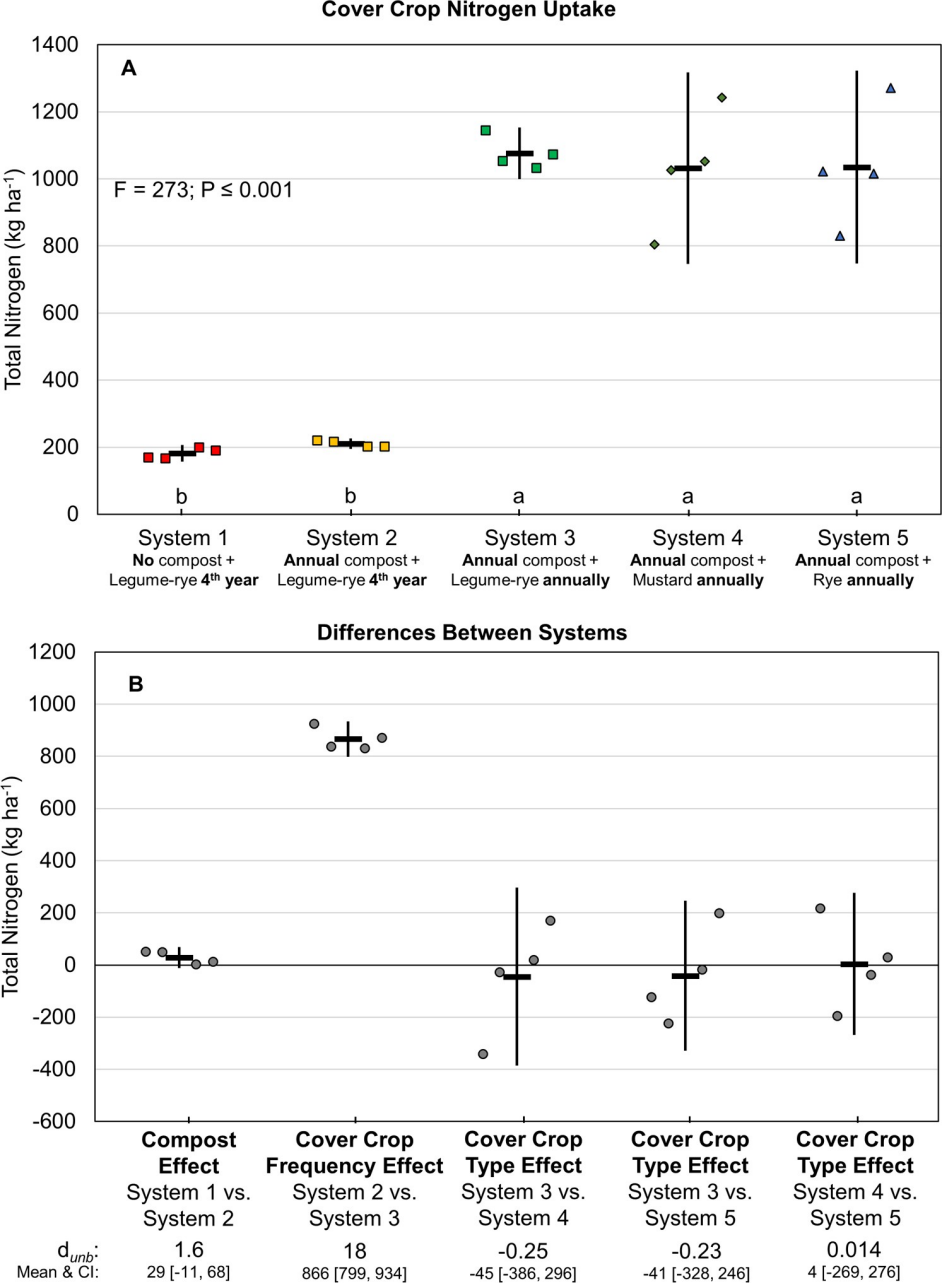
Cumulative above ground and estimated below ground cover crop nitrogen uptake over eight years of vegetable production. Mean nitrogen uptake (A) and mean paired differences between systems (B) in 5 organic vegetable systems. Systems differed by annual compost additions, cover crop type and cover cropping frequency. Nitrogen uptake data does not include nitrogen from legume nitrogen fixation in Systems 1, 2 and 3; see Table 3 for nitrogen fixation data. Error bars are 95% confidence intervals (CI) around the mean (horizontal line). Data points are replicates 1 through 4 averaged across years and are clustered around the mean left to right so as not to obscure the mean and CI. Different lower-case letters above the x-axis in plot A indicate that means are significantly different based on the Tukey-Kramer adjusted family-wise error rate of P≤0.05. The standardized effect size (Cohen’s unbiased *d*, *d*_*unb*_) is shown below the x-axis in plot B.

Cumulative N uptake—excluding N fixation—by the cover crops ranged from approximately 200 kg ha^-1^ when cover crops were planted quadrennially, to a little more than 1000 kg ha^-1^ when they were planted annually (Fig 5). Uptake was similar among the annually planted legume-rye, mustard and rye cover crops. In the three legume-rye systems, cover cropping frequency was the only factor that had a clear effect on N uptake, as indicated by the comparison of Systems 2 and 3 (*d*_unb_ = 18; Fig 5B). Estimated cumulative N fixation over the 8 years of vegetable production—which is not included in our uptake measurements—averaged 131 kg N ha^-1^ for Systems 1 and 2, and 424 kg N ha^-1^ System 3 (Table 3, S1 Fig).

**Table 3:** Cumulative nitrogen inputs, exports and mass balances for 5 organic vegetable systems over eight years in Salinas, CA. Systems differed by annual compost additions (0 vs. 7.6 Mg ha^-1^, oven-dry basis, before each vegetable crop), cover crop type (legume-rye, mustard, or cereal rye alone) and cover cropping frequency (quadrennially vs. annually planted). System 1 = no compost+legume-rye 4^th^ year; System 2 = annual compost+legume-rye 4^th^ year; System 3 = annual compost+legume-rye annually; System 4 = annual compost+mustard annually; System 5 = annual compost+rye annually.

	System 1		System 2		System 3		System 4		System 5	
*Inputs*	*kg N ha* ^*-1*^	*[95% CI]*	*kg N ha* ^*-1*^	*[95% CI]*	*kg N ha* ^*-1*^	*[95% CI]*	*kg N ha* ^*-1*^	*[95% CI]*	*kg N ha* ^*-1*^	*[95% CI]*
Atmospheric Deposition	3	─	3	─	3	─	3	─	3	─
Irrigation Water	155	─	155	─	155	─	155	─	155	─
Compost	0	─	1710	─	1710	─	1710	─	1710	─
Fertilizer^1^	1532	─	1532	─	1532	─	1532	─	1532	─
Vegetable Transplant Mix	14	─	14	─	14	─	14	─	14	─
Cover Crop Seed	32	─	32	─	127	─	4	─	19	─
Cover Crop N Fixation	133b	[113, 157]	129b	[119, 139]	424 a	[284, 564]	0	─	0	─
Total Inputs	1869	─	3575	─	3965	─	3418	─	3433	─
Lettuce Harvest Export	109d	[100, 119]	136c	[117, 155]	202 a	[189, 215]	174b	[156, 192]	173b	[154, 193]
Broccoli Harvest Export	320d	[301, 339]	338cd	[295, 382]	455 a	[392, 518]	418ab	[338, 498]	381bc	[314, 448]
Total Harvest Exports	429c	[415, 444]	474c	[412, 536]	657 a	[603, 711]	592ab	[494, 689]	554bc	[472, 635]
**Soil Surface Balance**^**2**^	**1440**d	[1412, 1468]	**3101**b	[3047, 3156]	**3308** a	[3099, 3442]	**2826**c	[2729, 2924]	**2879**c	[2798, 2961]
Change in Soil N Storage^3^	0	─	662a	[268, 1056]	936 a	[690, 1182]	830a	[–6, 1665]	891a	[470, 1311]
**Soil System Balance**^**4**^	**1440**b	[1412, 1468]	**2439**a	[1996, 2883]	**2372** a	[2124, 2620]	**1996**a	[1065, 2926]	**1988**a	[1527, 2450]
(surplus to 30 cm)										
% Total Inputs Exported										
in Harvest	23a	[21, 24]	13c	[12, 15]	16 b	[15, 18]	17b	[16, 19]	16b	[15, 18]
Fertilizer N Input: Export	3.6a	[3.3, 3.8]	3.2a	[3.0, 3.5]	2.3 c	[2.1, 2.6]	2.6bc	[2.4, 2.9]	2.8b	[2.5, 3.0]
Total N Input: Export	4.4c	[3.8, 5.0]	7.5a	[6.9, 8.1]	6.0 b	[5.4, 6.6]	5.8b	[5.3, 6.4]	6.2b	[5.7, 6.8]

Means in the same row with the same letter are not significantly different based on the Tukey-Kramer adjusted family-wise error rate of P≤0.05.

^1^Applied in pelleted and liquid organic fertilizers.

^2^Difference between total nitrogen inputs and total nitrogen exported in harvested vegetables.

^3^Difference in mean total nitrogen stock (Fig 2) for a given System relative to System 1.

^4^Difference between the Soil Surface Balance and Change in Soil Nitrogen Storage

**Fig 6:**
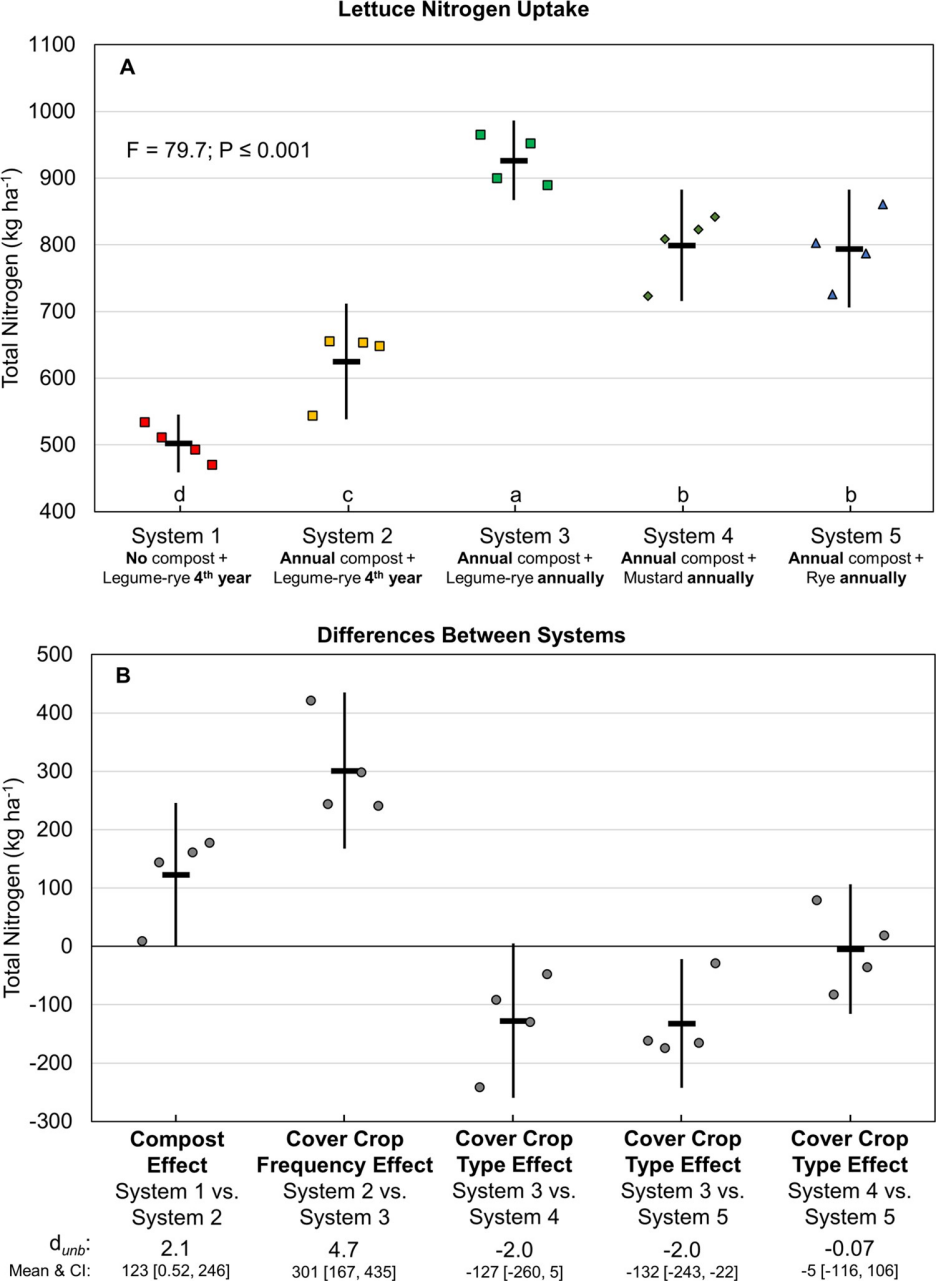
Cumulative above ground and estimated below ground lettuce nitrogen uptake over eight years of vegetable production. Mean nitrogen uptake (A) and mean paired differences between systems (B) in 5 organic vegetable systems. Systems differed by annual compost additions, cover crop type and cover cropping frequency. Error bars are 95% confidence intervals (CI) around the mean (horizontal line). Data points are replicates 1 through 4 averaged across years and are clustered around the mean left to right so as not to obscure the mean and CI. Different lower-case letters above the x-axis in plot A indicate that means are significantly different based on the Tukey-Kramer adjusted family-wise error rate of P≤0.05. The standardized effect size (Cohen’s unbiased *d*, *d*_*unb*_) is shown below the x-axis in plot.

In lettuce—the first vegetable crop following cover crop incorporation or fallow—increased cover cropping frequency had more than double the effect of compost on crop N uptake, which is evident when comparing the standard effect size of cover crop frequency vs. compost (*d*_unb_ = 4.7 vs. 2.1; Fig 6B). Compared with System 1 (no compost + cover cropping every 4^th^ year), lettuce N uptake increased by 123 kg ha^-1^ in System 2 (with compost), and an additional 301 kg ha^-1^ following annual legume-rye cover cropping in System 3 (Fig 6). Nitrogen uptake by lettuce was similar in Systems 4 and 5 following mustard or rye cover crops, respectively, but averaged 130 kg ha^-1^ lower than in System 3 following the legume-rye cover crop.

**Fig 7:**
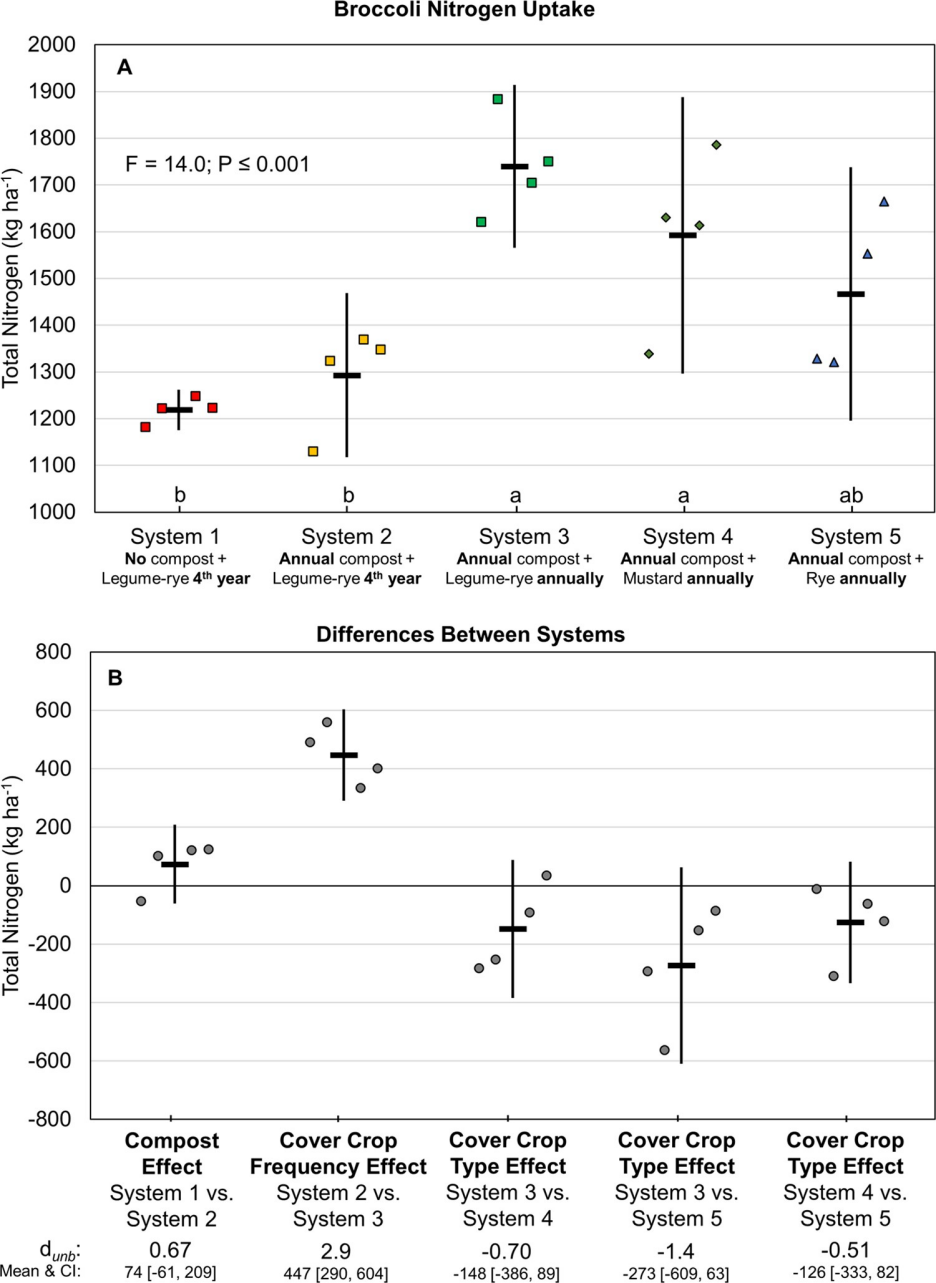
Cumulative above ground and estimated below ground broccoli nitrogen uptake over six years of vegetable production. Mean cumulative nitrogen uptake (A) and mean paired differences between systems (B) in 5 organic vegetable systems. Systems differed by annual compost additions, cover crop type and cover cropping frequency. Error bars are 95% confidence intervals (CI) around the mean (horizontal line). Data points are replicates 1 through 4 averaged across years and are clustered around the mean left to right so as not to obscure the mean and CI. Different lower-case letters above the x-axis in plot A indicate that means are significantly different based on the Tukey-Kramer adjusted family-wise error rate of P≤0.05. The standardized effect size (Cohen’s unbiased *d*, *d*_*unb*_) is shown below the x-axis in plot B.

Increased cover crop frequency also increased broccoli N uptake (Fig 7), which followed a similar but less pronounced pattern as that for lettuce (*d*_unb_ = 2.9; Fig 7B; vs *d*_unb_ = 4.7; Fig 6B). Annual legume-rye cover crops in System 3 increased broccoli cumulative N uptake by 447 kg ha^-1^ compared to quadrennial cover cropped System 2. Although the 95% CIs of all the other system comparisons for broccoli N uptake overlapped with zero (Fig 7B), the direction of all these comparisons is consistent with the patterns in lettuce N uptake, with the exception of System 4 (mustard) versus System 5 (rye) (Fig 6B).

### Vegetable yields and residue nitrogen

**Fig 8:**
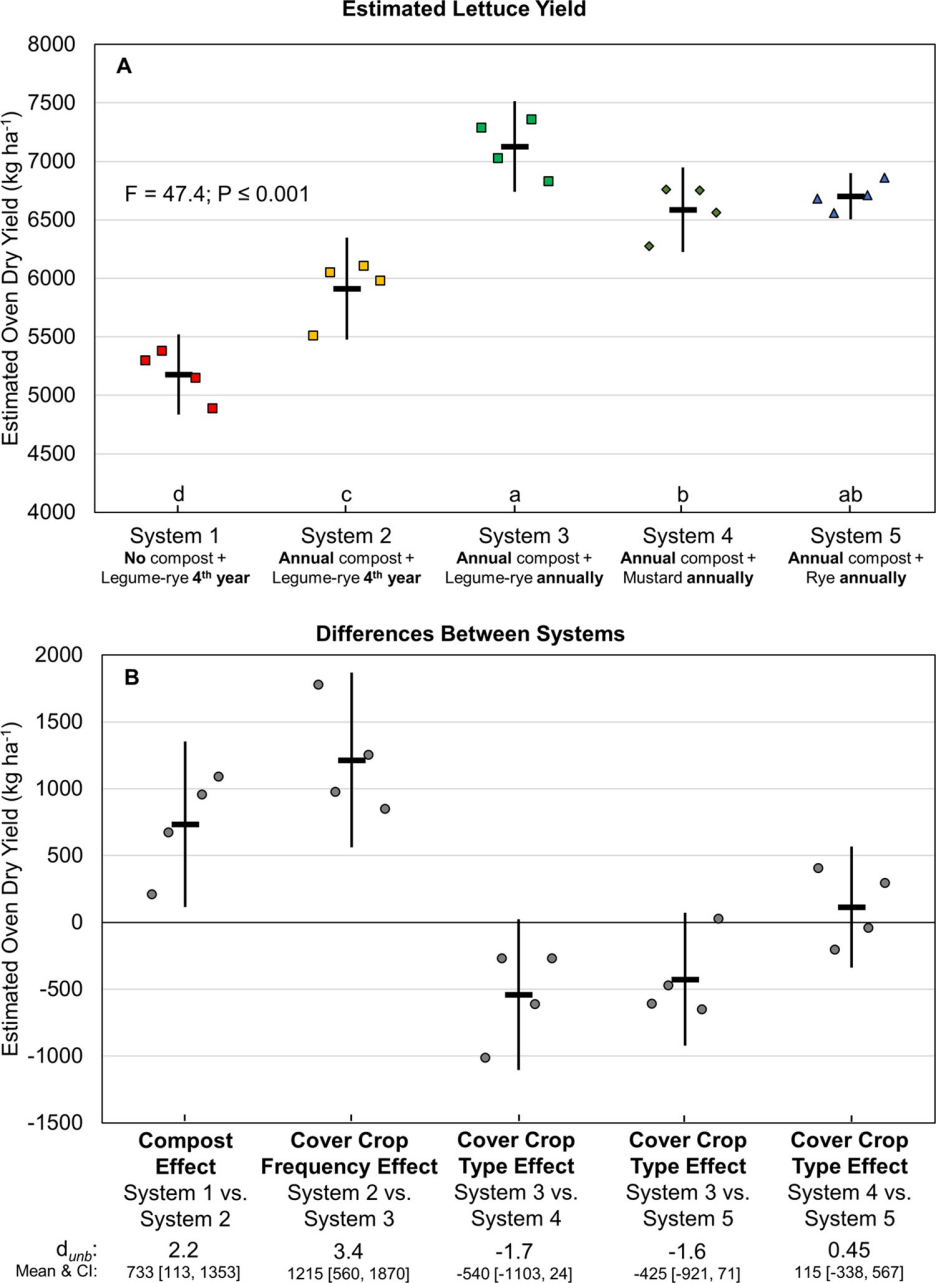
Cumulative, estimated, marketable lettuce yield over eight years. Mean cumulative lettuce yield (A) and mean paired differences between systems (B) in 5 organic vegetable systems. Yields are oven-dry biomass. Systems differed by annual compost additions, cover crop type and cover cropping frequency. Error bars are 95% confidence intervals (CI) around the mean (horizontal line). Data points are replicates 1 through 4 averaged across years and are clustered around the mean left to right so as not to obscure the mean and CI. Different lower-case letters above the x-axis in plot A indicate that means are significantly different based on the Tukey-Kramer adjusted family-wise error rate of P≤0.05. The standardized effect size (Cohen’s unbiased *d*, *d*_*unb*_) is shown below the x-axis in plot B.

**Fig 9:**
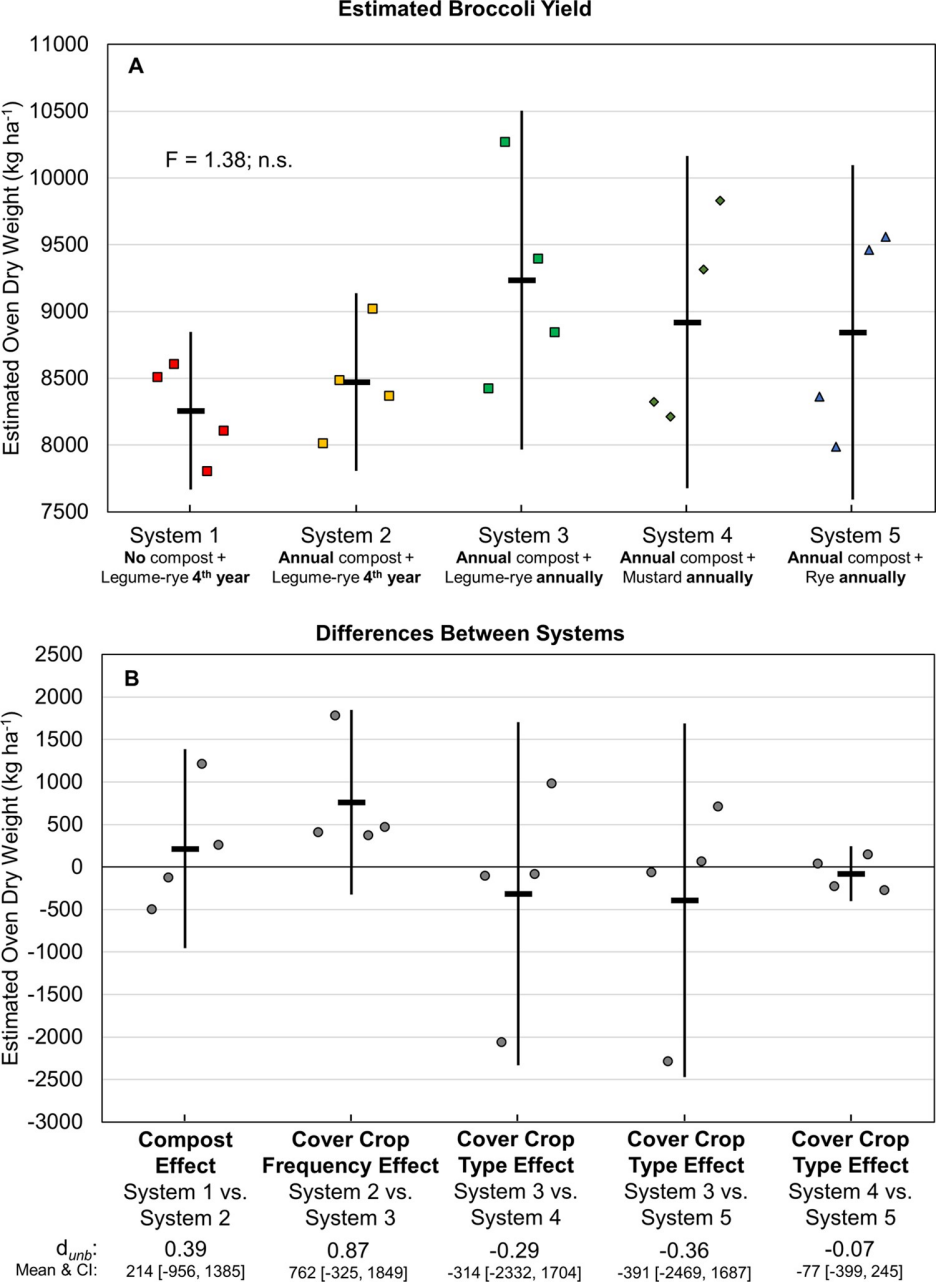
Cumulative, estimated, marketable broccoli yield over six years. Mean cumulative broccoli yield (A) and mean paired differences between systems (B) in 5 organic vegetable systems. Yields are oven-dry biomass. Systems differed by annual compost additions, cover crop type and cover cropping frequency. Error bars are 95% confidence intervals (CI) around the mean (horizontal line). Data points are replicates 1 through 4 averaged across years and are clustered around the mean left to right so as not to obscure the mean and CI. The standardized effect size (Cohen’s unbiased *d*, *d*_*unb*_) is shown below the x-axis in plot B. Broccoli was only grown during 6 of the first 8 years of the experiment.

Mean cumulative, estimated, marketable yields of lettuce over eight years ranged from 5180 to 7128 kg ha^-1^; and for six years of broccoli from 8258 to 9235 kg ha^-1^ (Figs 8 and 9, respectively). Mean lettuce yield in the legume-rye cover cropped systems was in order of System 1 < System 2 < System 3. Broccoli followed a similar pattern, although yields were more variable. Increased legume-rye cover crop frequency boosted lettuce yields by 1215 kg ha^-1^ (Systems 2 vs 3, *d*_unb_ = 3.4; Fig 8B); the same general pattern occurred with broccoli, although the CI of the effect overlapped with zero (Fig 9B). Compost increased lettuce yields by 733 kg ha^-1^ (Fig 8B), but did not have a consistent effect on broccoli yields as illustrated by the scatter of the raw data and the large overlap of the CI with 0 (Fig 9B). Among the annually cover cropped systems, vegetable yields tended to be higher following legume-rye than mustard or rye (Figs 8 and 9).

**Fig 10:**
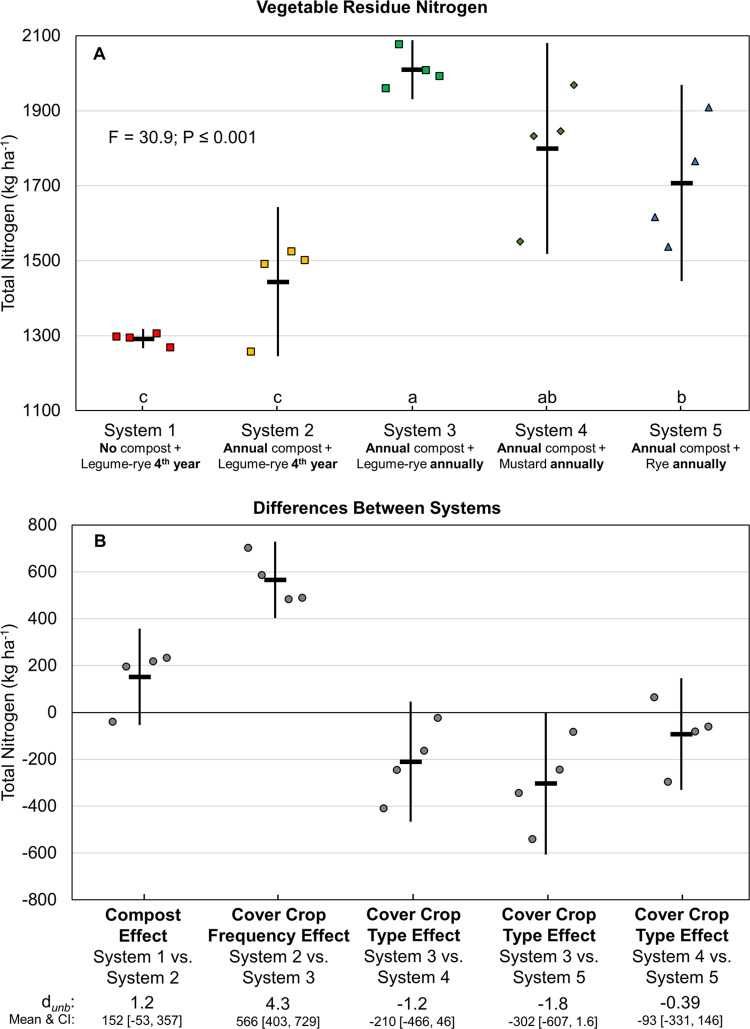
Cumulative above ground and estimated below ground vegetable residue nitrogen over eight years of vegetable production. Mean cumulative vegetable (lettuce + broccoli) residue nitrogen (A) and mean paired differences between systems (B) in 5 organic vegetable systems. Systems differed by annual compost additions, cover crop type and cover cropping frequency. Error bars are 95% confidence intervals (CI) around the mean (horizontal line). Data points are replicates 1 through 4 averaged across years and are clustered around the mean left to right so as not to obscure the mean and CI. Different lower-case letters above the x-axis in plot A indicate that means are significantly different based on the Tukey-Kramer adjusted family-wise error rate of P≤0.05. The standardized effect size (Cohen’s unbiased *d*, *d*_*unb*_) is shown below the x-axis in plot B.

Vegetable residue N returned to the soil after harvest ranged from 1292 to 2010 kg ha^-1^ over 8 years (Fig 10). Vegetable residue N followed the patterns observed for vegetable N uptake and yield, increasing or tending to increase with compost and annual cover cropping. Increased vegetable biomass due to greater N uptake resulted in 566 kg ha^-1^ greater vegetable residue N due to increased legume-rye cover crop frequency in System 3 compared to System 2 (*d*_unb_ = 4.3; Fig 10B.)

### Nitrogen balances

**Fig 11:**
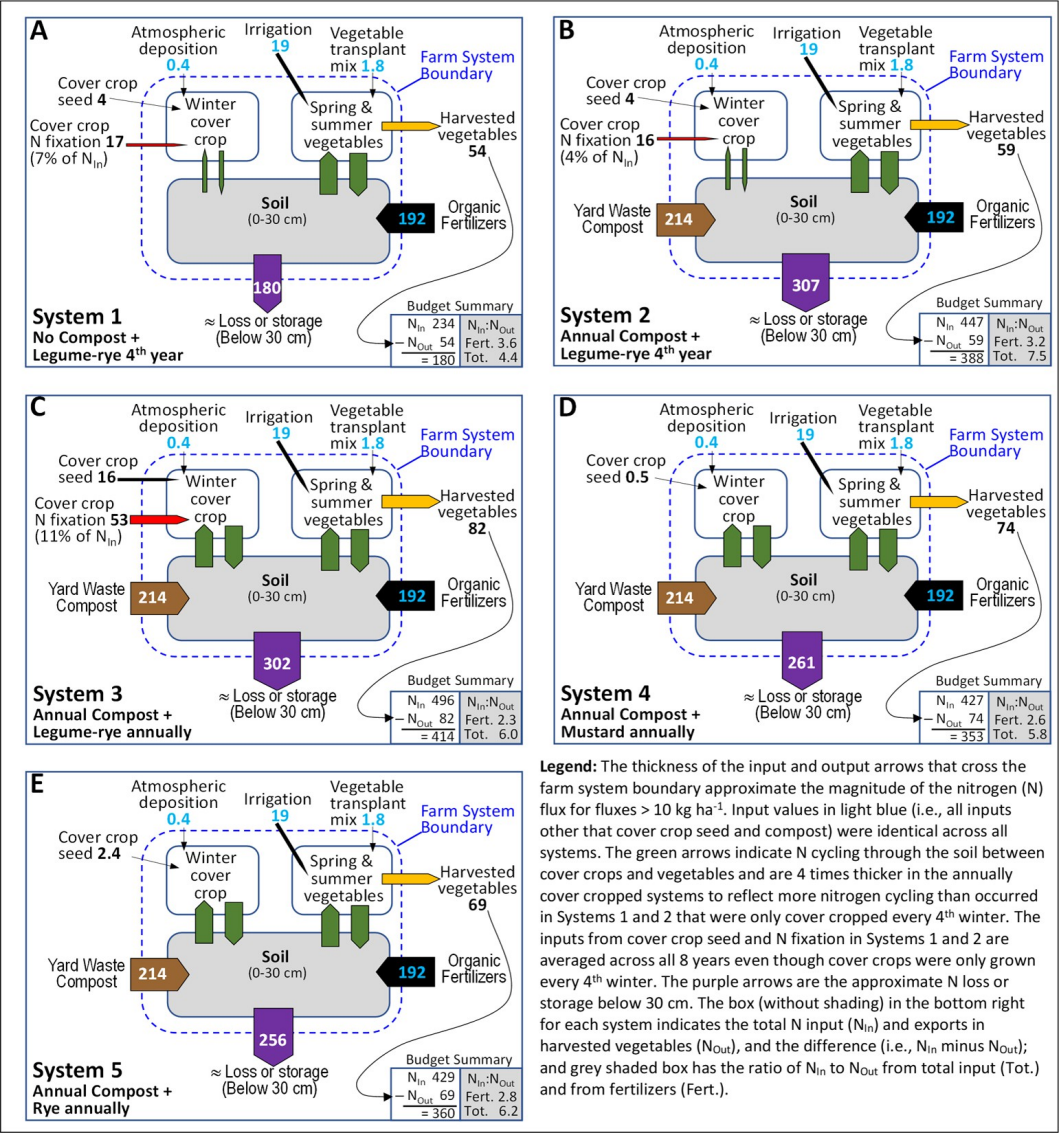
Average annual nitrogen inputs, exports and storage or losses in five organic vegetable systems over 8 years. Values are in kg ha^-1^ and were calculated from the cumulative values in Table 2. Systems differed by annual compost additions, cover crop type and cover cropping frequency.

**Fig 12:**
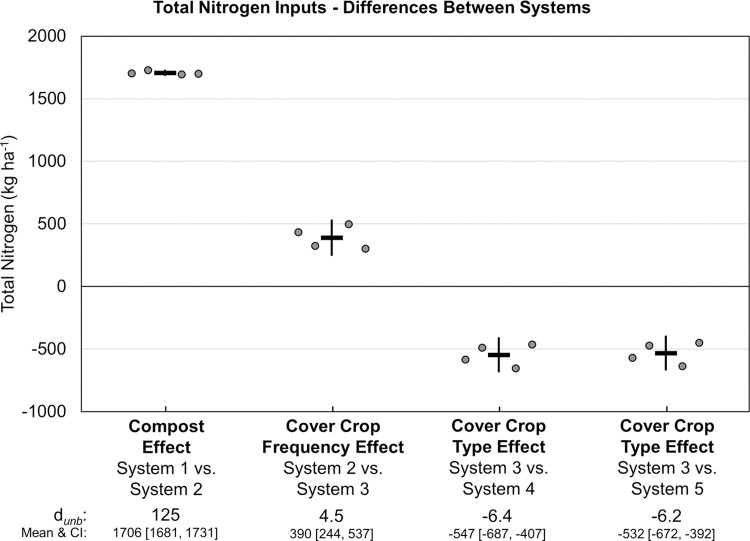
Mean paired difference in cumulative total nitrogen inputs between systems over eight years of vegetable production. Measurements were taken in five organic vegetable systems. Systems differed by annual compost additions, cover crop type and cover cropping frequency. System 1 = no compost+legume-rye 4^th^ year; System 2 = annual compost+legume-rye 4^th^ year; System 3 = annual compost+legume-rye annually; System 4 = annual compost+mustard annually; System 5 = annual compost+rye annually. The difference between Systems 4 and 5 is not presented because all inputs were fixed, thus there was no variation among replicates. Error bars are 95% confidence intervals (CI) around (horizontal line). Data points are replicates 1 through 4 averaged across years and are clustered around the mean left to right so as not to obscure the mean and CI. The standardized effect size (Cohen’s unbiased *d*, *d*_*unb*_) is shown below the x-axis.

Annual N inputs, particularly from organic fertilizers and/or compost exceeded exports in harvested vegetables in all systems by at least 4-fold (Fig 11, Table 3). Over 8 years, all five systems received identical inputs from atmospheric deposition (3 kg ha^-1^), vegetable transplant mix (14 kg ha^-1^), irrigation water (155 kg ha^-1^), and organic fertilizers (1532 kg ha^-1^) (Table 3). Nitrogen inputs that differed among systems included cover crop seed (4 to 127 kg ha^-1^), N fixation (0 to 424 kg ha^-1^) and compost (0 or 1710 kg ha^-1^). Consequently, total N inputs ranged from 1869 kg ha^-1^ in System 1 to 3418 to 3965 kg ha^-1^ in the four systems receiving compost. Compost had the largest effect on N inputs (*d*_unb_ = 125; Fig 12). Differences among the four systems that received compost were due to cover crop frequency and the presence of legume cover crops that influenced N inputs from cover crop seed and N fixation (Fig 11B–11E, Table 3).

**Fig 13:**
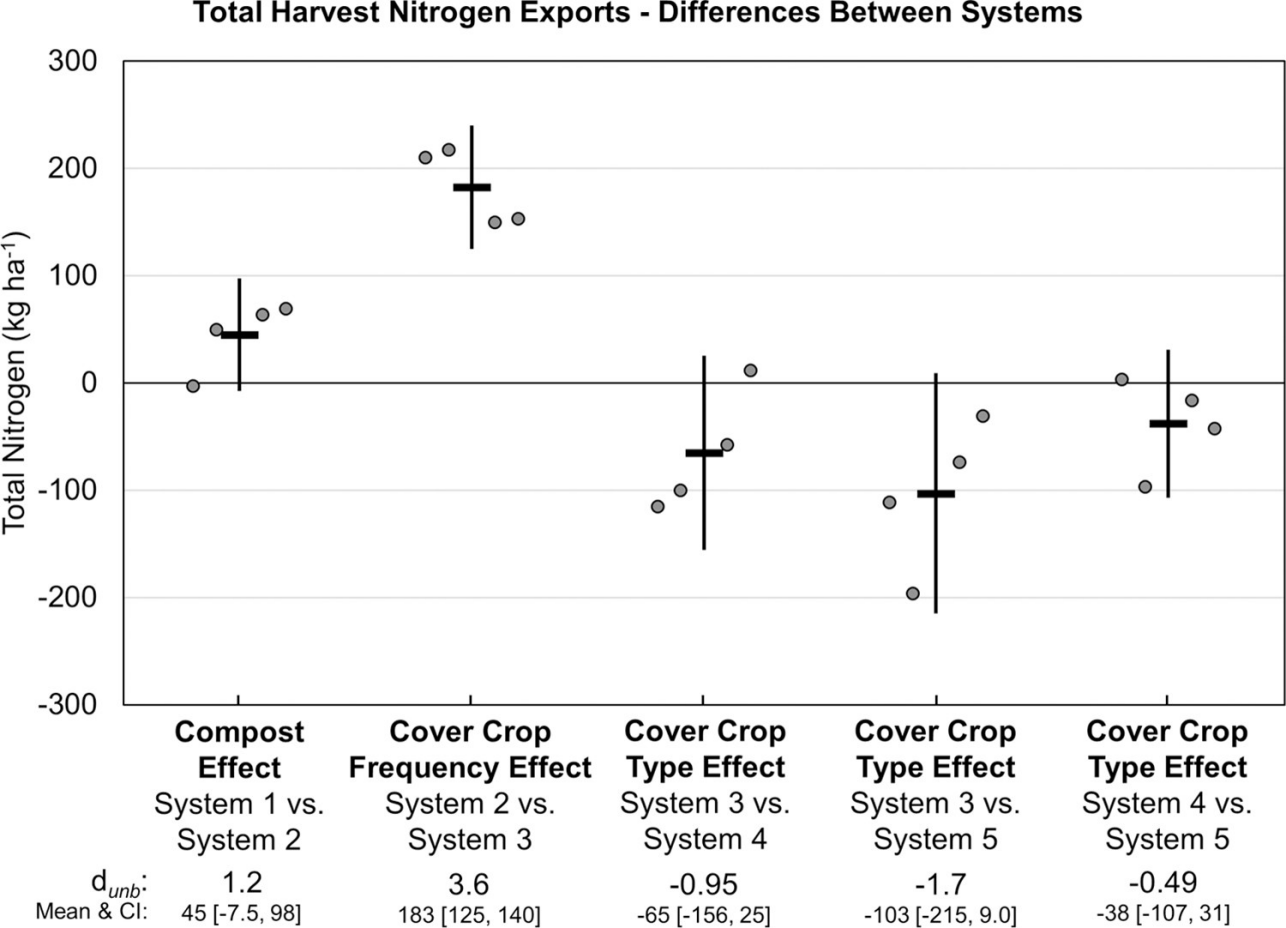
Mean paired difference in cumulative total harvest nitrogen exports between systems over eight years of vegetable production. Measurements were taken in five organic vegetable systems. Systems differed by annual compost additions, cover crop type and cover cropping frequency. System 1 = no compost+legume-rye 4^th^ year; System 2 = annual compost+legume-rye 4^th^ year; System 3 = annual compost+legume-rye annually; System 4 = annual compost+mustard annually; System 5 = annual compost+rye annually. Error bars are 95% confidence intervals (CI) around the mean (horizontal line). Data points are replicates 1 through 4 averaged across years and are clustered around the mean left to right so as not to obscure the mean and CI. The standardized effect size (Cohen’s unbiased *d*, *d*_*unb*_) is shown below the x-axis.

**Fig 14:**
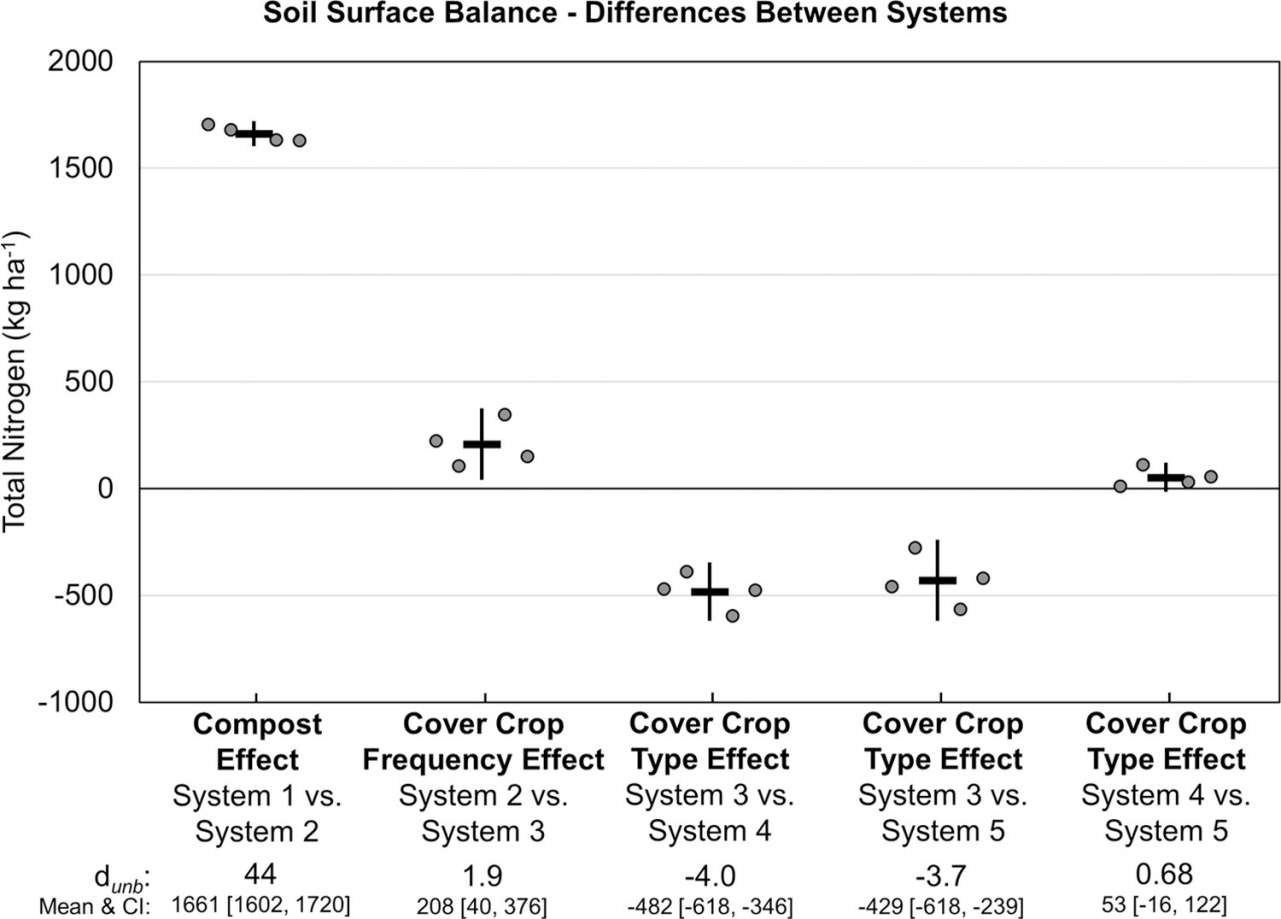
Mean paired difference in cumulative soil surface nitrogen balance between systems over eight years of vegetable production. Measurements were taken in five organic vegetable systems. Systems differed by annual compost additions, cover crop type and cover cropping frequency. System 1 = no compost+legume-rye 4^th^ year; System 2 = annual compost+legume-rye 4^th^ year; System 3 = annual compost+legume-rye annually; System 4 = annual compost+mustard annually; System 5 = annual compost+rye annually. Error bars are 95% confidence intervals (CI) around the mean (horizontal line). Data points are replicates 1 through 4 averaged across years and are clustered around the mean left to right so as not to obscure the mean and CI. The standardized effect size (Cohen’s unbiased *d*, *d*_*unb*_) is shown below the x-axis.

The ratio of total N inputs to harvest N exports ranged from 4.4 (23% of inputs exported) in System 1 to 7.5 (13% of inputs exported) in System 2 (Fig 11A and 11B). Greater N uptake and yield among the three annually cover cropped systems led to correspondingly greater N export in harvested crops than in quadrennially cover cropped systems, resulting in intermediate ratios of total N input to N export for Systems 3, 4 and 5 (Fig 11C–11E). Compared with quadrennial cover cropping, annual legume-rye cover crops increased harvest N exports by 183 kg ha^-1^ (*d*_unb_ = 3.6; Fig 13). Consequently, the soil surface balance (i.e., N inputs minus harvest N exports) ranged from 1440 kg ha^-1^ in System 1 to between 2826 to 3308 kg ha^-1^ in the annually cover cropped systems receiving compost. Annual legume-rye cover cropping increased the soil surface N balance by 208 kg ha^-1^ (i.e., Systems 2 versus 3, *d*_unb_ = 1.9; Fig 14).

**Fig 15:**
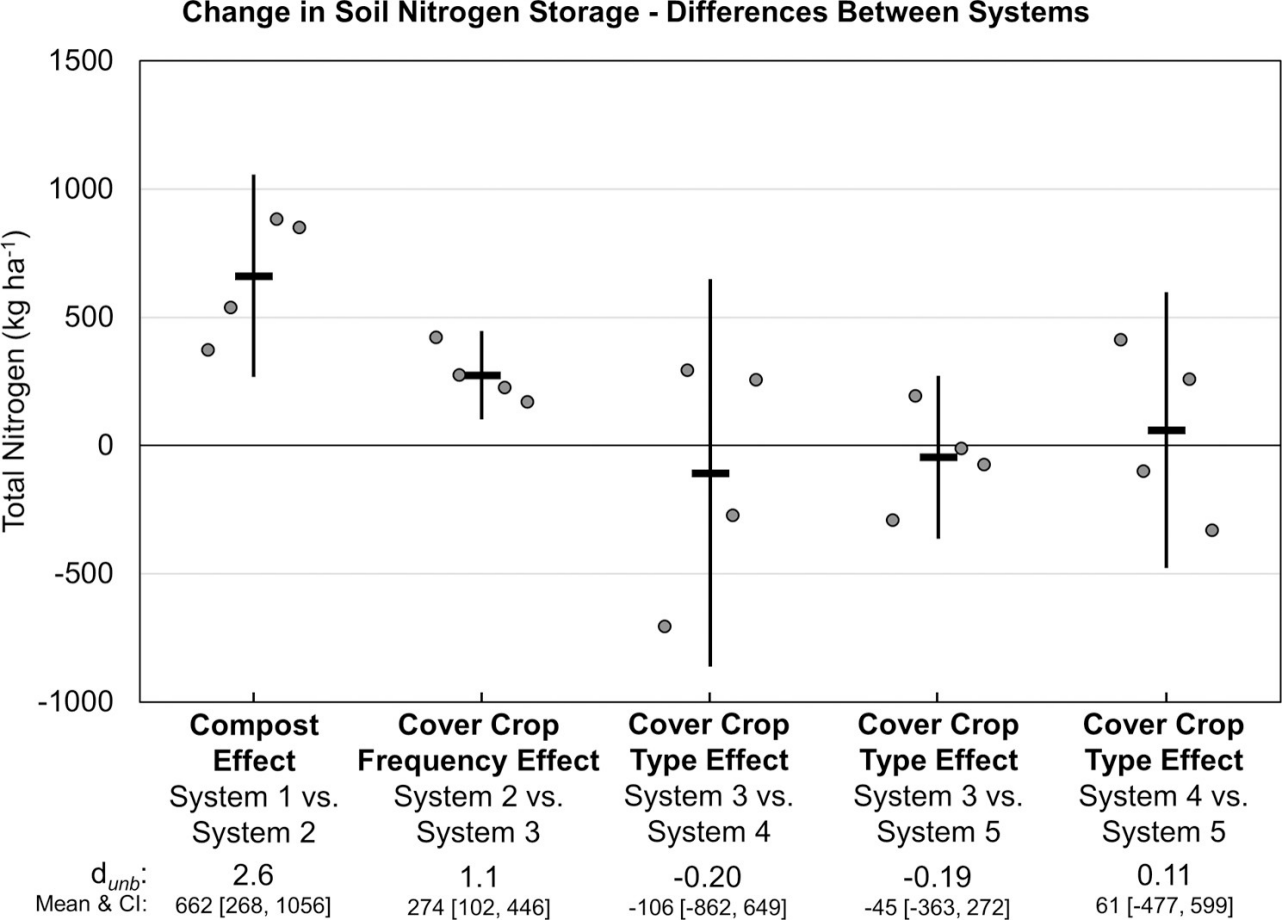
Mean paired difference in cumulative soil nitrogen storage change between systems over eight years of vegetable production. Measurements were taken in five organic vegetable systems. Systems differed by annual compost additions, cover crop type and cover cropping frequency. System 1 = no compost+legume-rye 4^th^ year; System 2 = annual compost+legume-rye 4^th^ year; System 3 = annual compost+legume-rye annually; System 4 = annual compost+mustard annually; System 5 = annual compost+rye annually. Error bars are 95% confidence intervals (CI) around the mean (horizontal line). Data points are replicates 1 through 4 averaged across years and are clustered around the mean left to right so as not to obscure the mean and CI. The standardized effect size (Cohen’s unbiased *d*, *d*_*unb*_) is shown below the x-axis.

**Fig 16:**
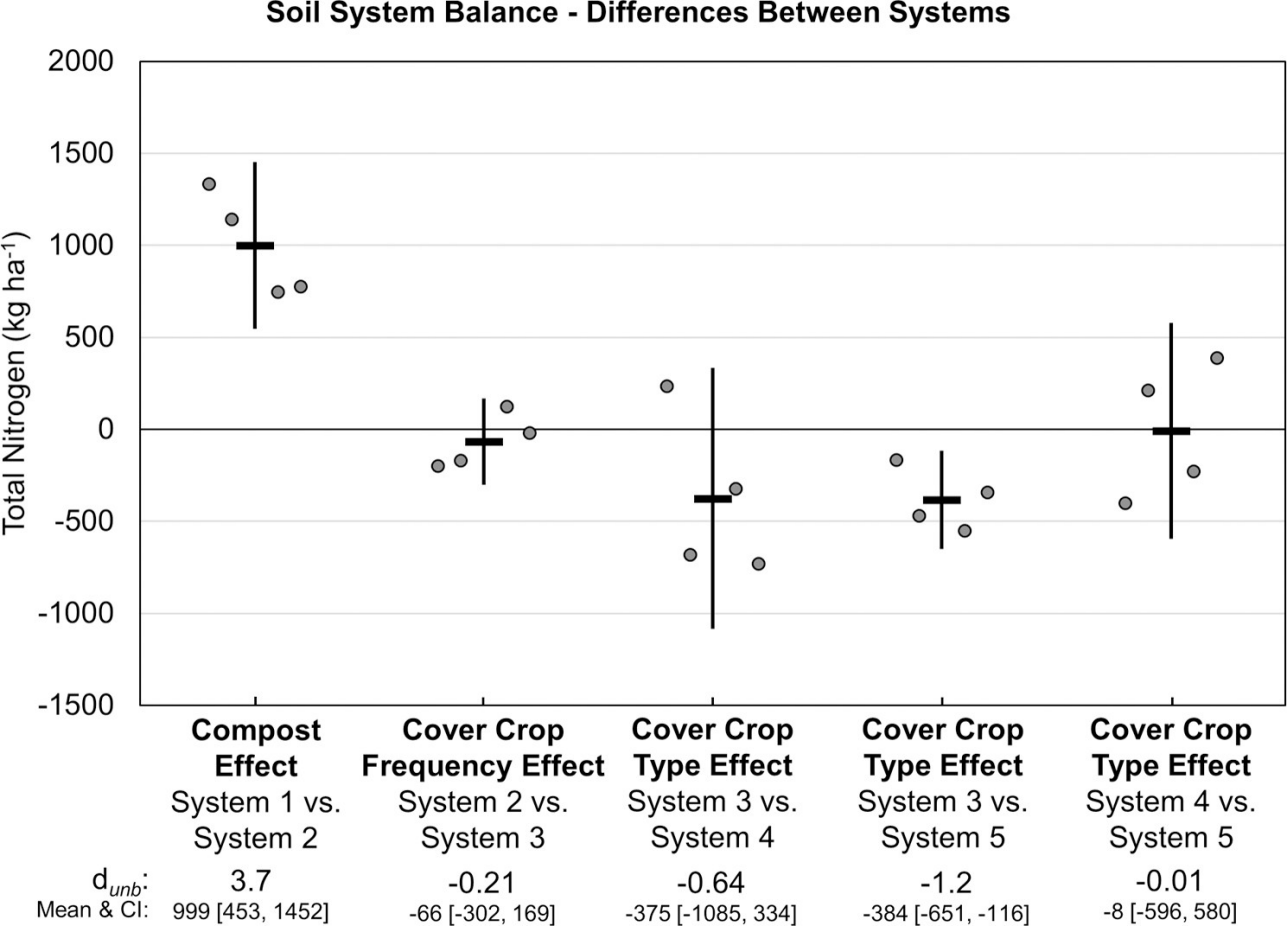
Mean paired difference in cumulative soil system nitrogen balance between systems over eight years of vegetable production. Measurements were taken in five organic vegetable systems. Systems differed by annual compost additions, cover crop type and cover cropping frequency. System 1 = no compost+legume-rye 4^th^ year; System 2 = annual compost+legume-rye 4^th^ year; System 3 = annual compost+legume-rye annually; System 4 = annual compost+mustard annually; System 5 = annual compost+rye annually. Error bars are 95% confidence intervals (CI) around the mean (horizontal line). Data points are replicates 1 through 4 averaged across years and are clustered around the mean left to right so as not to obscure the mean and CI. The standardized effect size (Cohen’s unbiased *d*, *d*_*unb*_) is shown below the x-axis.

The change in soil N storage (i.e., System 2, 3, 4 or 5 N stocks minus System 1 N stocks) was primarily influenced by compost application, which increased soil N stocks by 662 kg ha^-1^ (*d*_unb_ = 2.6; Fig 15). Subtracting soil N storage (i.e., the difference in mean TN stock for each system relative to System 1) from their respective soil surface balances improved N balances as indicated by lower soil system relative to soil surface N balances. Compost also had the largest impact on soil system N balances (*d*_unb_ = 3.7; Fig 16). While increased cover crop frequency did not have an effect on soil system N balances (Fig 16), annual mustard and rye cover crops, which do not fix atmospheric N, reduced the N surplus by an average of 380 kg ha^-1^ in Systems 4 and 5 compared to that in System 3 over eight years (Table 3).

## Discussion

This is the first study on the long-term effects of compost and cover crops on soil N balances, and their relationships to crop performance in tillage-intensive, organic, high-value vegetable systems in California. Our results suggest that N balances in these systems could be improved substantially with no to minimal impact on crop yields by reducing compost additions and using non-leguminous cover crops annually.

### Nitrogen dynamics

#### Initial decline in N stocks

Across all systems, soil N stocks declined by approximately 50% from year 0 to year 1 (Fig 1), following the same pattern as for soil C stocks [43]. These declines were likely due to a transition from relatively low intensity cropping in the years before the experiment to the intensive practices (i.e., more tillage, fertilization, frequent inputs of fresh plant residue, and irrigation) that began in year 1 and continued through year 8 [47]. Others reported that increasing tillage in soils receiving organic amendments increases readily mineralizable soil N and mineralization rates [75, 76]. In a Georgia tomato production system, total N stocks declined 20% following the first year of moldboard plowing on a sandy loam soil despite incorporation of a hairy vetch cover crop and fertilizer application of 180 kg N ha^-1^ [77].

#### Inefficient use of N

In our experiment, N inputs applied at rates typical for the region were used inefficiently with only 13 to 23% of total inputs exported in harvested vegetables (Table 3). Fertilizer N application was 2.3 to 3.6 times greater than harvest exports and this imbalance was exacerbated by N inputs from compost and biological fixation by legumes. Presumably, only a fraction of pelleted organic fertilizer inputs were mineralized and available for uptake during the cropping cycle [18]. Though this mineralized N may be utilized efficiently by crop plants, a sizeable proportion of the total fertilizer N input (45 to 65%) remains unmineralized in soil following crop harvest [18]. In addition, much of the N taken up by crop plants was ultimately returned to the soil in residues rather than exported during harvest. Given the relatively mild winters in the region, N mineralization from soil organic matter and crop residues occurs all year and the risk of winter NO_3_-N leaching in Salinas systems is well documented [23, 78, 79]. Without annual winter cover crops to intercept plant available N, losses of N in Systems 1 and 2 were undoubtedly higher than in Systems 3, 4 and 5 that were cover cropped annually. For example, another study in the Salinas valley, found that winter NO_3_-N leached after conventional broccoli was 25.5 g m^-2^ under bare fallow and only 7.4 g m^-2^ with a rye cover crop [23].

#### Challenges caused by compost

Compost was the main contributor to the N surpluses in Systems 2, 3, 4 and 5 (Table 3), but had little effect on lettuce N uptake and yield (Figs 6 and 8) and had practically no effect on broccoli N uptake and yield (Figs 7 and 9); for example, compost added 214 kg N ha^-1^ annually but only increased annual lettuce N uptake by about 15 kg ha^-1^ (Fig 6). Likewise, large annual applications of compost, in addition to organic fertilizer, resulted in 81% greater N inputs relative to crop exports in Brazilian organic leafy vegetable production [4]. Short-term (< 84 days) laboratory incubations in silty loam soil indicate that N availability from yard waste compost is limited, and likely explains why compost had little effect on crop yield in our study [18]. Farmers in the Salinas valley typically consider compost with a C:N ratio greater than 11 as a soil amendment to improve soil physical characteristics, not as a short-term source of N [80]. As such, compost is applied in California vegetable production systems at rates comparable to those in our study to build soil organic matter and enhance the ability of the soil to supply N and other nutrients over the long term. However, the increase in System 2 soil N storage in the top 30 cm from compost accounted for only 39% of compost N inputs (Table 3, 662 kg N ha^-1^ storage/1710 kg N ha^-1^ input from compost), suggesting that much of the remaining added compost N was lost via leaching and, or, denitrification or stored in soil below 30 cm (discussed below). In less intense cropping systems on finer textured soils in the Sacramento Valley of California, fields where compost was the primary organic input had higher soil C and N storage and apparently better temporal synchrony between soil N availability and plant uptake [28]. However, in our experiment, the small effect of compost on vegetable N uptake (Figs 6 and 7) suggests that compost N mineralization was not well synchronized with vegetable crop N demand. Furthermore, the relative similarity and stability of N stocks from year 1 onwards in Systems 2, 3, 4, and 5 (Figs 1B, 1C, 1D and 1E) reveal that there was no long-term impact of annual compost applications on soil N storage; this finding runs counter to the general scientific consensus and expectations of regional experts (Brennan and Smith) who were involved with the study design. The difference between our results and observations on finer textured soils elsewhere in CA [28], demonstrates the strong role of soil texture in influencing C and N dynamics. We are not aware of published reports of the prevalence of compost use in this region, but in two of the authors’ experience it is the predominant practice for adding organic matter with the goal of improving soil health because it can be readily integrated in tight crop production schedules [31].

#### Benefits from cover crops

In contrast to annual compost additions, the effects of cover crops were generally positive and consistent with previous results from this study. For example, annual cover cropping increased soil enzyme activities [52], N retention in labile soil C (POX-C) [47], and microbial biomass C and N [51]; cover crops also had the greatest effect on the soil food web, as indicated by the structure and functions of the nematode assemblage [81]. As a result, annual cover cropping supported greater soil N mineralization and availability, and thus increased fertilizer N use efficiency (Table 3), crop N uptake (Figs 6 and 7), lettuce yields (Fig 8), and harvest N exports (Table 3, Fig 12); in addition, by intercepting N mineralized from decomposing vegetable crop residues, annual cover cropping also facilitated cycling of N from one year to the next. We are unaware of other similar long-term studies, but a 2-year field study in Japan found that a legume-rye cover crop increased lettuce N uptake by 36% and yields by 22 to 27% [82]. Furthermore, a 2-year study in the Salinas valley found that the positive effects of a legume-oat (*Avena sativa* L.) cover crop on broccoli N uptake and yield were variable and dependent on fertilizer application rate, cover crop C:N and site management history [83]. While the legume-rye cover crop in System 3 of our study generally had the largest positive effect on fertilizer N use efficiency, crop yield, and N export–compared with the mustard in System 4 and the rye in System 5 –these benefits were offset somewhat by the increase in total N inputs from legume N fixation. This increased the N balances in System 3 relative to the non-legume cover cropped systems, and thus increased the risk of N leaching losses; this is apparent in the season-end nitrate concentrations that were usually highest in System 3 (Figs 3 and 4). In contrast, by cycling previously applied N, the annual non-legume cover crops also increased the efficient use of existing N and subsequent vegetable yields and N exports but did so without increasing total N inputs. Our results demonstrate that additional N inputs beyond crop fertilizer requirements are not retained in these intensively managed systems.

#### What was the fate of the excess N inputs?

The fate of excess N inputs is an important concern raised by our research. While a portion of this N was stored in the top 30 cm of soil, the majority was either transported below 30 cm or lost to the environment. We assume that runoff and erosion losses were minimal on this sandy soil that was leveled to a slope of approximately 1%. Given the excellent drainage and likely infrequent anaerobic conditions in the soil at the research site, we assume that gaseous losses were minimal. Although laboratory incubations of yard waste compost show relatively low annual mineralization rates (i.e., < 2.5% of total N) and suggest that compost N poses a low risk to leaching [18], our data do not support this conclusion. We suspect that in the sandy soil and field conditions in our study—high tillage intensity coupled with diverse inputs from compost, organic fertilizers and decomposing cover crop and vegetable crop residues—resulted in much greater N mineralization rates than those measured in laboratory incubations [18]. We suspect that these conditions resulted in a priming effect [84, 85], in which increased activity of soil microorganisms specialized in degrading readily available inputs also used the more recalcitrant C from compost as substrate [86]. The resulting pool of mineralized NO_3_-N and dissolved organic N [87] would be vulnerable to leaching in the sandy soils at the study site. These losses would be particularly acute during bare winter fallows in Systems 2. Additional research is necessary to test this hypothesis by measuring leaching losses in these systems.

Excessive irrigation can cause N leaching during dry spring and summer periods when most vegetable production occurs in this region [79]. However, a study with ^15^N-labelled cover crop residues on the same soil as in our experiment found that leaching to 60 cm during spring and summer vegetables was relatively small when irrigation scheduling matched crop water demand [78]. We carefully scheduled vegetable irrigation in our study based on soil moisture and ET data from a nearby weather station of the California Irrigation Management Information System [88], and therefore we doubt that irrigation caused appreciable N leaching. We also used vegetable transplants, that develop root systems relatively quickly and have higher initial N demand, rather the direct seeding the vegetables. Therefore, leaching losses in our systems most likely occurred when vegetables were not growing (Oct/Nov-Feb/March) when rainfall could exceed ET by the cover crops.

Non-legume cover crops and mixtures of non-legume and legume cover crops can reduce winter NO_3_-N leaching losses by approximately 50 to 70% [23, 29, 89]. However, few studies have measured leaching of dissolved organic N, which can be substantial in sandy soils [90, 91]. Cover crop roots often extend below 1 m [30, 92] and thus may capture some of the N leached below our 30 cm soil system boundary and recycle it for later vegetable uptake. Broccoli roots in Salinas Valley fields also often extend below 30 cm and thus may help to capture N leached deeper into the soil profile during the growing season [93]. Nevertheless, the large N surpluses in our study suggest high N leaching losses in all systems, regardless of cover crop use, which indicates a crucial need to improve N management in these systems to protect water quality in the region. While our study reflects a range of regional organic management scenarios, relatively few organic farms in the Salinas Valley cover crop each field annually (E. Brennan, personal communication).

### Strategies to improve nitrogen management

Our results are consistent with those of other researchers who have studied nutrient flows in organic systems [11, 12] and reveal that current fertilization strategies in these high input, intensive organic vegetable production systems need to be improved. We provide a few suggestions.

#### Eliminate compost inputs

Eliminating compost applications would reduce the soil surface budget surpluses by 50 to 60%, with the greatest reductions in Systems 4 and 5, which included non-legume cover crops. However, without compost applications or annual cover crops, soil surface budget surpluses in System 1 still left 180 kg N ha^-1^ y^-1^ potentially available for leaching loss. However, eliminating compost applications would reduce soil C stocks [47] and forgo the potential soil health benefits provided by compost applied to coarse textured soils [94]. Despite the potential benefits of compost, it is important to highlight that during the first 6 years of our study, the change in soil health from annual inputs of compost–as indicated by microbial biomass C–was relatively small when cover crops were only grown every 4^th^ winter (System 2: 40 mg C kg^-1^ soil) compared to when they were grown every winter (System 3: 147 mg C kg^-1^ soil) [51].

#### Non-legume cover crops rather than legume-cereal mixes

Among the annually cover cropped systems, non-legume cover crops in Systems 4 and 5 resulted in the lowest N balances due to the absence of N fixation by legumes and lower N inputs in cover crop seed. Potential N leaching losses in these systems—as indicated by post-harvest soil NO_3_-N concentrations—were often reduced compared to those in the legume-rye System 3. In a hypothetical non-legume cover cropped system without compost application–assuming the same cover crop effect on soil N storage and harvest N exports presented in Table 3 –the annual soil system N surpluses in Systems 4 and 5 would average 118 kg ha^-1^ y^-1^, less than the yearly N uptake by cover crops (131 kg ha^-1^ y^-1^) in Systems 3, 4 and 5 (Fig 5). In comparison, yearly N surpluses would be greater (166 kg ha^-1^ y^-1^) relative to cover crop N uptake due to System 3 legume N inputs. Though some proportion of N leaching losses may have occurred after vegetable harvest and prior to peak cover crop N uptake in the fall, annual non-legume cover crops limit this loss.

While vegetable yields in the non-legume cover crop-based systems were slightly lower compared to those in the annual legume-rye based System 3, non-legume cover crops are a considerably more cost-effective strategy to improve N balances. The seed cost for the legume-rye mixture in System 3 ($680 ha^-1^) was almost ten times greater than for mustard ($72 ha^-1^) and rye ($69 ha^-1^) in Systems 4 and 5, respectively [56]. The non-seed costs to plant and manage a cover crop in this region are typically about $250 ha^-1^ [95]. Consequently, vegetable farmers may be able to capture more leachable NO_3_-N in the winter on a whole farm basis if they spend their cover cropping budget on non-legume cover crop seed, thus allowing them to plant cover crops on more acres. However, because dissolved organic N is not taken up by plants in large quantities [96] cover cropping needs to be used in synchrony with reducing N inputs.

Two additional strategies to reduce fertilizer N inputs may be to adjust application rates, 1) to account for N fixation in systems with legume cover crops, and 2) based on potential soil N mineralization. However, given interannual variation in cover crop biomass production [83, 97] and the effect of management history on soil N mineralization [98], additional long-term research is necessary to develop improved management recommendations to reduce fertilizer application rates using these approaches.

### Limitations of the study

There are 4 important limitations of our study. First, we did not measure potential N leaching or denitrification losses; the latter pathway is unlikely given the course texture of our soil. Second, our study lacked systems that were cover cropped annually without compost; thus, we were not able to directly assess the effect of cover crops alone on N uptake and yield. Third, we were unable to assess the effects of cover cropping frequency in systems with non-legume cover crops because non-legume systems were only cover cropped annually. Non-legume cover crops like rye and mustard are often preferred in Salinas Valley vegetable production systems because, in contrast to some legumes, they are generally not considered hosts of soilborne diseases [99, 100] and seed costs are lower [56]. Fourth, the same amount of fertilizer was applied to all systems regardless of yields. Therefore, the N balances in Systems 1 and 2 likely underestimate the typical N inputs applied in infrequently cover cropped organic and conventional systems in this region.

## Conclusions

- The high soil surface N balances indicate that N inputs were used relatively inefficiently and were at high risk of N leaching.
- Organic fertilizers were an important N source for vegetables, but compost had little effect on vegetable yields.
- Annually planted cover crops increased N availability, uptake, and vegetable yield by improving nutrient retention and cycling.
- Lettuce yields were higher following a legume-rye cover crop than following a rye or mustard cover crop; but this effect was not apparent in the subsequent broccoli.
- Soil N stocks declined by about 50% during the first year of vegetable production in all systems and continued a gradual decline over time without compost in System 1.
- Compost (combined with cover crops) did not increase soil N stocks but maintained N stocks after the first year of vegetable production.
- Among systems receiving annual compost, N stocks trended higher in annually cover cropped systems compared to those with quadrennial cover cropping.
- Reduced reliance on imported amendments (e.g., compost) and increased reliance on on-farm produced organic matter inputs from cover crops could improve N availability, crop yield, and N balance surpluses in intensive vegetable production systems, and consequently reduce the risk of N leaching. However, this change will require novel approaches [101] to cover crop management.

## Supporting information

S1 Fig Cumulative cover crop nitrogen fixation over eight years of vegetable production. Estimates for taken in three organic vegetable systems in Salinas, CA which differed by cover cropping frequency (quadrennially vs. annually planted). System 1 = no compost+legume-rye 4^th^ year; System 2 = annual compost+legume-rye 4^th^ year; System 3 = annual compost+legume-rye annually. Error bars are 95% confidence intervals (CI) with the mean at the horizontal line. Individual data points are averaged across years for replicates 1 through 4 of each system and are clustered around the mean in order from left to right so that they do no obscure the mean and CI.
(TIF)

S2 Fig Cumulative harvest exports over eight years of vegetable production. Measurements were taken in five organic vegetable systems in Salinas, CA. Systems differed by annual compost additions (0 vs.7.6 Mg ha before each vegetable crop, oven-dry basis), cover crop type (legume-rye, mustard, or cereal rye alone) and cover cropping frequency (quadrennially vs. annually planted). System 1 = no compost+legume-rye 4^th^ year; System 2 = annual compost+legume-rye 4^th^ year; System 3 = annual compost+legume-rye annually; System 4 = annual compost+mustard annually; System 5 = annual compost+rye annually. Error bars are 95% confidence intervals (CI) with the mean at the horizontal line. Individual data points are averaged across years for replicates 1 through 4 of each system and are clustered around the mean in order from left to right so that they do no obscure the mean and CI.
(TIF)

S3 Fig Mean soil surface nitrogen balance between systems over eight years of vegetable production. Measurements were taken in five organic vegetable systems in Salinas, CA. Systems differed by annual compost additions (0 vs.7.6 Mg ha before each vegetable crop, oven-dry basis), cover crop type (legume-rye, mustard, or cereal rye alone) and cover cropping frequency (quadrennially vs. annually planted). System 1 = no compost+legume-rye 4^th^ year; System 2 = annual compost+legume-rye 4^th^ year; System 3 = annual compost+legume-rye annually; System 4 = annual compost+mustard annually; System 5 = annual compost+rye annually. Error bars are 95% confidence intervals (CI) with the mean at the horizontal line. Individual data points are averaged across years for replicates 1 through 4 of each system and are clustered around the mean in order from left to right so that they do no obscure the mean and CI.
(TIF)

S4 Fig Mean soil nitrogen storage change between systems over eight years of vegetable production. Measurements were taken in five organic vegetable systems in Salinas, CA. Systems differed by annual compost additions (0 vs.7.6 Mg ha before each vegetable crop, oven-dry basis), cover crop type (legume-rye, mustard, or cereal rye alone) and cover cropping frequency (quadrennially vs. annually planted). System 1 = no compost+legume-rye 4th year; System 2 = annual compost+legume-rye 4th year; System 3 = annual compost+legume-rye annually; System 4 = annual compost+mustard annually; System 5 = annual compost+rye annually. Error bars are 95% confidence intervals (CI) with the mean at the horizontal line. Individual data points are averaged across years for replicates 1 through 4 of each system and are clustered around the mean in order from left to right so that they do no obscure the mean and CI.
(TIF)

S5 Fig Mean soil system nitrogen balance between systems over eight years of vegetable production. Measurements were taken in five organic vegetable systems in Salinas, CA. Systems differed by annual compost additions (0 vs.7.6 Mg ha before each vegetable crop, oven-dry basis), cover crop type (legume-rye, mustard, or cereal rye alone) and cover cropping frequency (quadrennially vs. annually planted). System 1 = no compost+legume-rye 4^th^ year; System 2 = annual compost+legume-rye 4^th^ year; System 3 = annual compost+legume-rye annually; System 4 = annual compost+mustard annually; System 5 = annual compost+rye annually. Error bars are 95% confidence intervals (CI) with the mean at the horizontal line. Individual data points are averaged across years for replicates 1 through 4 of each system and are clustered around the mean in order from left to right so that they do no obscure the mean and CI.
(TIF)
