# Supplementary figures and images for: Winter cover crops increased nitrogen availability and efficient use during eight years of intensive organic vegetable production
Source: PLoS One. 2022 Apr 28;17(4):e0267757. doi: 10.1371/journal.pone.0267757 (PMC9049554; doi:10.1371/journal.pone.0267757)

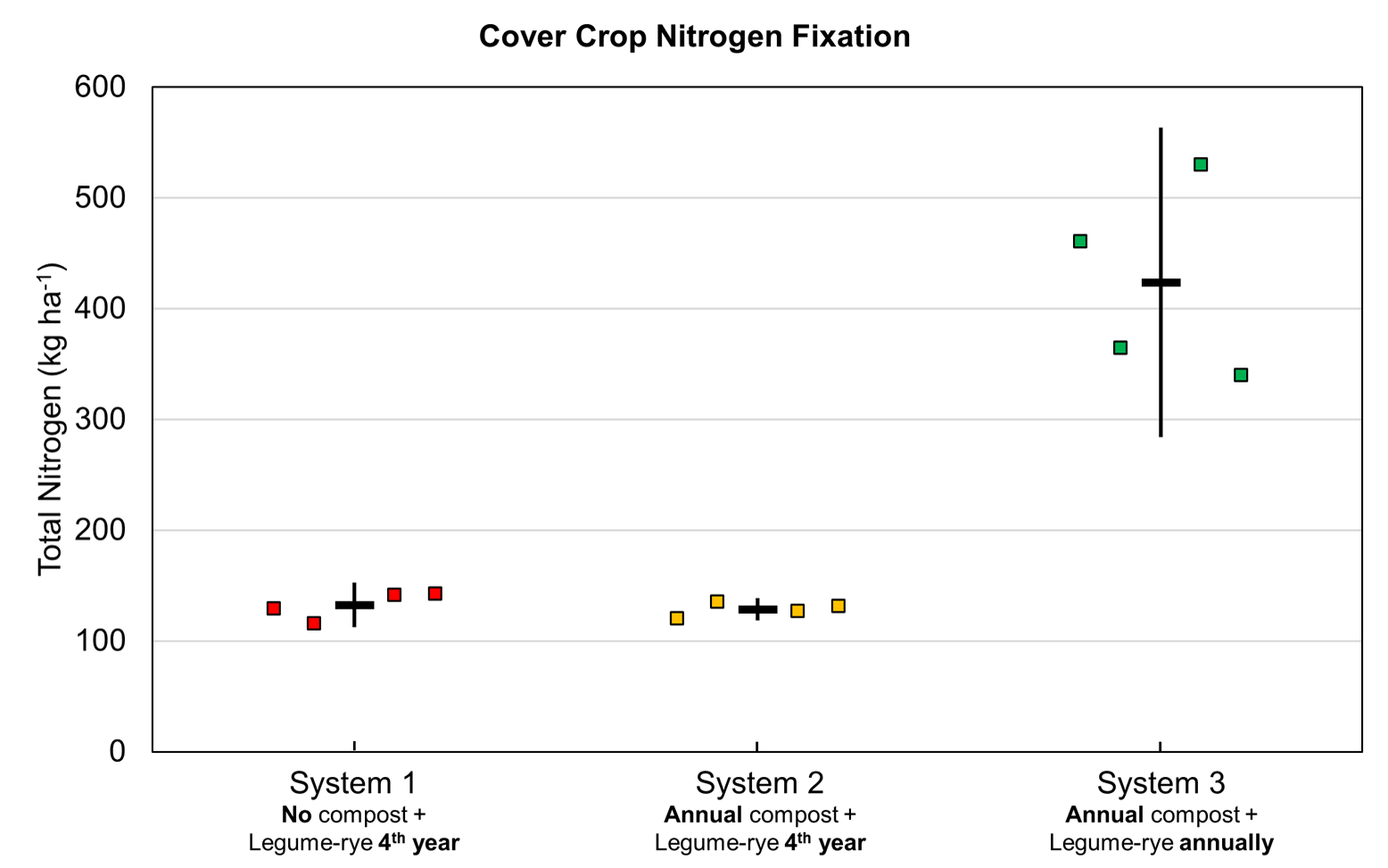

Supplement: S1 Fig — Estimates for taken in three organic vegetable systems in Salinas, CA which differed by cover cropping frequency (quadrennially vs. annually planted). System 1 = no compost+legume-rye 4th year; System 2 = annual compost+legume-rye 4th year; System 3 = annual compost+legume-rye annually. Error bars are 95% confidence intervals (CI) with the mean at the horizontal line. Individual data points are averaged across years for replicates 1 through 4 of each system and are clustered around the mean in order from left to right so that they do no obscure the mean and CI. (TIF) [file pone.0267757.s001.tif]

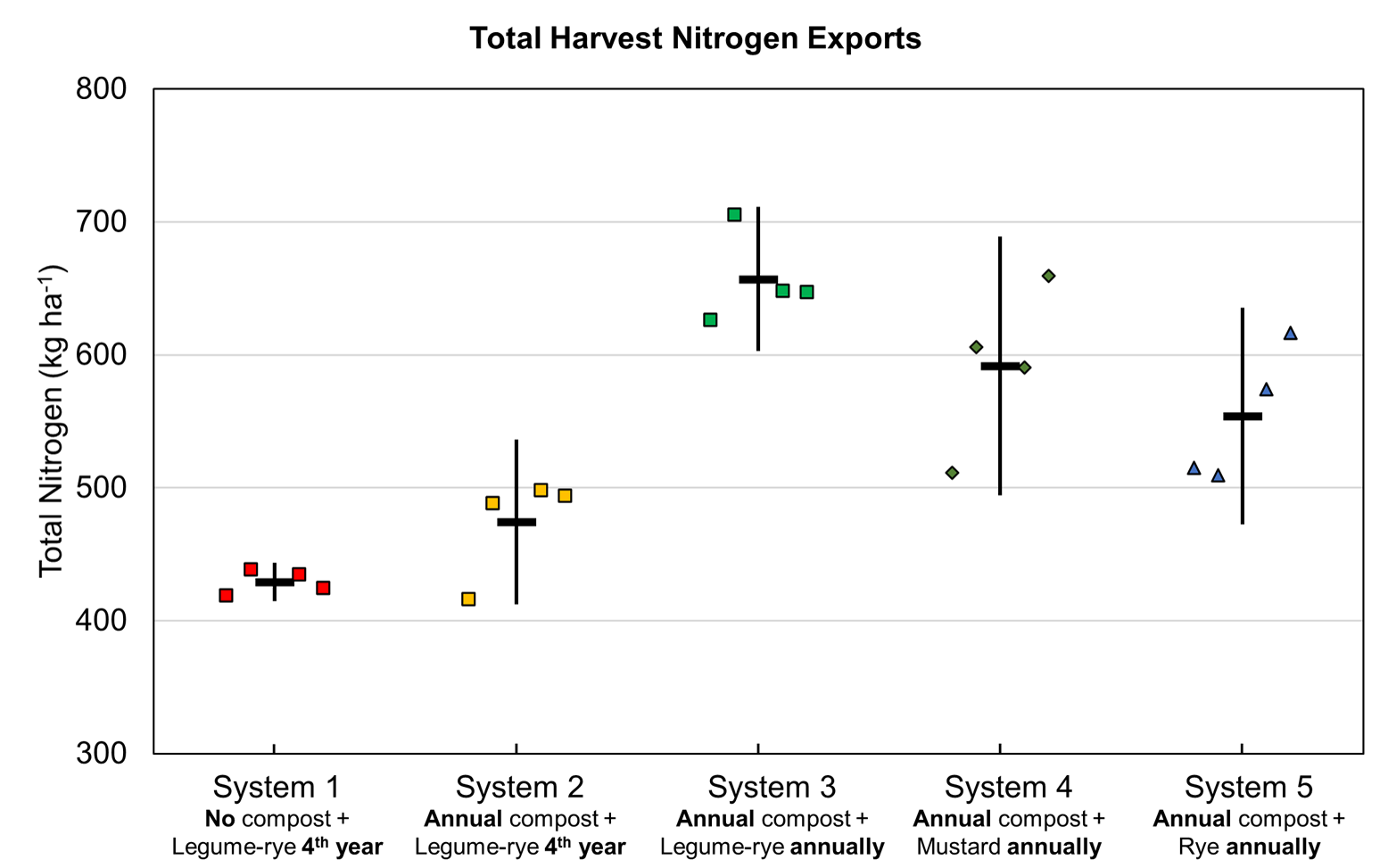

Supplement: S2 Fig — Measurements were taken in five organic vegetable systems in Salinas, CA. Systems differed by annual compost additions (0 vs.7.6 Mg ha before each vegetable crop, oven-dry basis), cover crop type (legume-rye, mustard, or cereal rye alone) and cover cropping frequency (quadrennially vs. annually planted). System 1 = no compost+legume-rye 4th year; System 2 = annual compost+legume-rye 4th year; System 3 = annual compost+legume-rye annually; System 4 = annual compost+mustard annually; System 5 = annual compost+rye annually. Error bars are 95% confidence intervals (CI) with the mean at the horizontal line. Individual data points are averaged across years for replicates 1 through 4 of each system and are clustered around the mean in order from left to right so that they do no obscure the mean and CI. (TIF) [file pone.0267757.s002.tif]

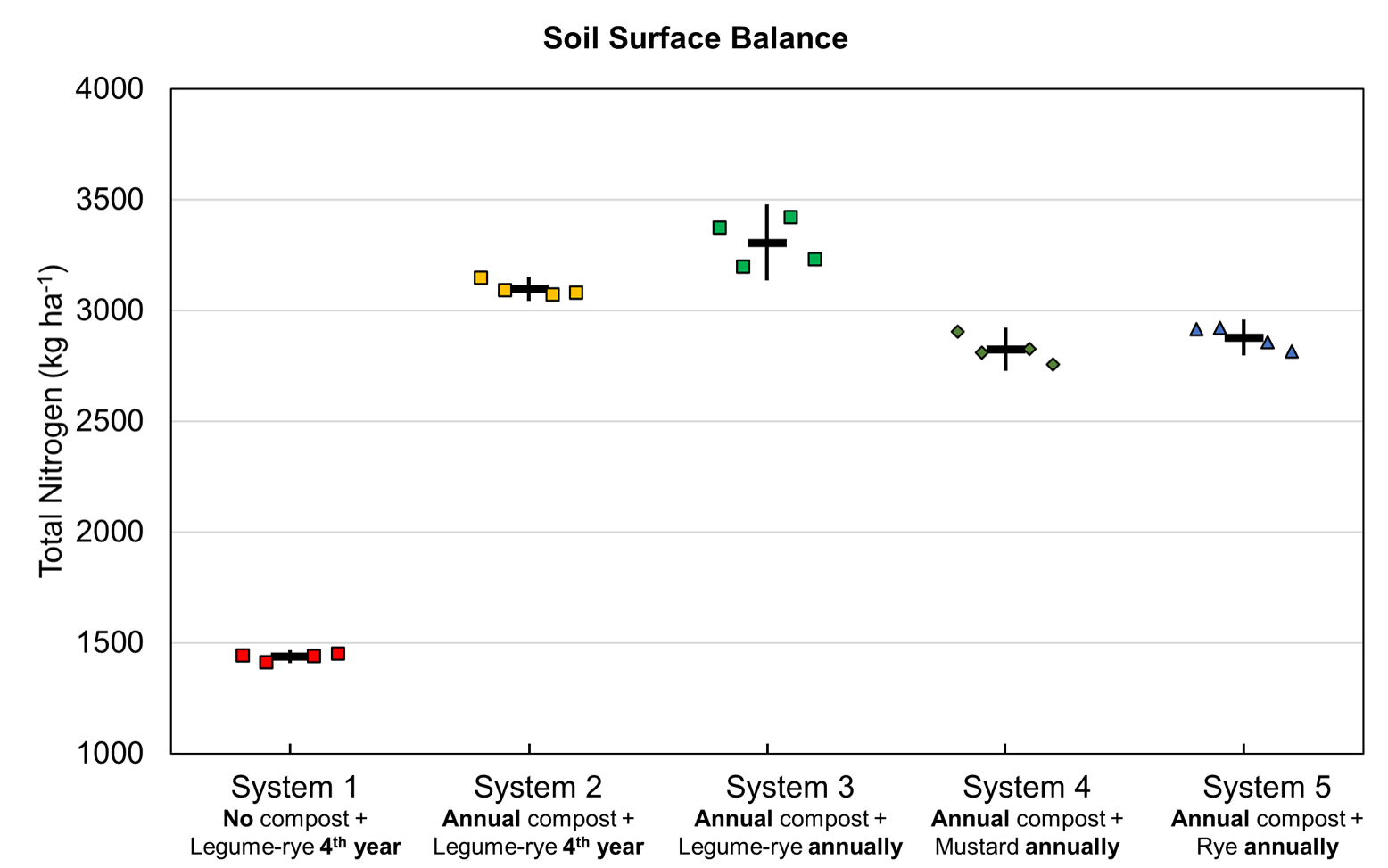

Supplement: S3 Fig — Measurements were taken in five organic vegetable systems in Salinas, CA. Systems differed by annual compost additions (0 vs.7.6 Mg ha before each vegetable crop, oven-dry basis), cover crop type (legume-rye, mustard, or cereal rye alone) and cover cropping frequency (quadrennially vs. annually planted). System 1 = no compost+legume-rye 4th year; System 2 = annual compost+legume-rye 4th year; System 3 = annual compost+legume-rye annually; System 4 = annual compost+mustard annually; System 5 = annual compost+rye annually. Error bars are 95% confidence intervals (CI) with the mean at the horizontal line. Individual data points are averaged across years for replicates 1 through 4 of each system and are clustered around the mean in order from left to right so that they do no obscure the mean and CI. (TIF) [file pone.0267757.s003.tif]

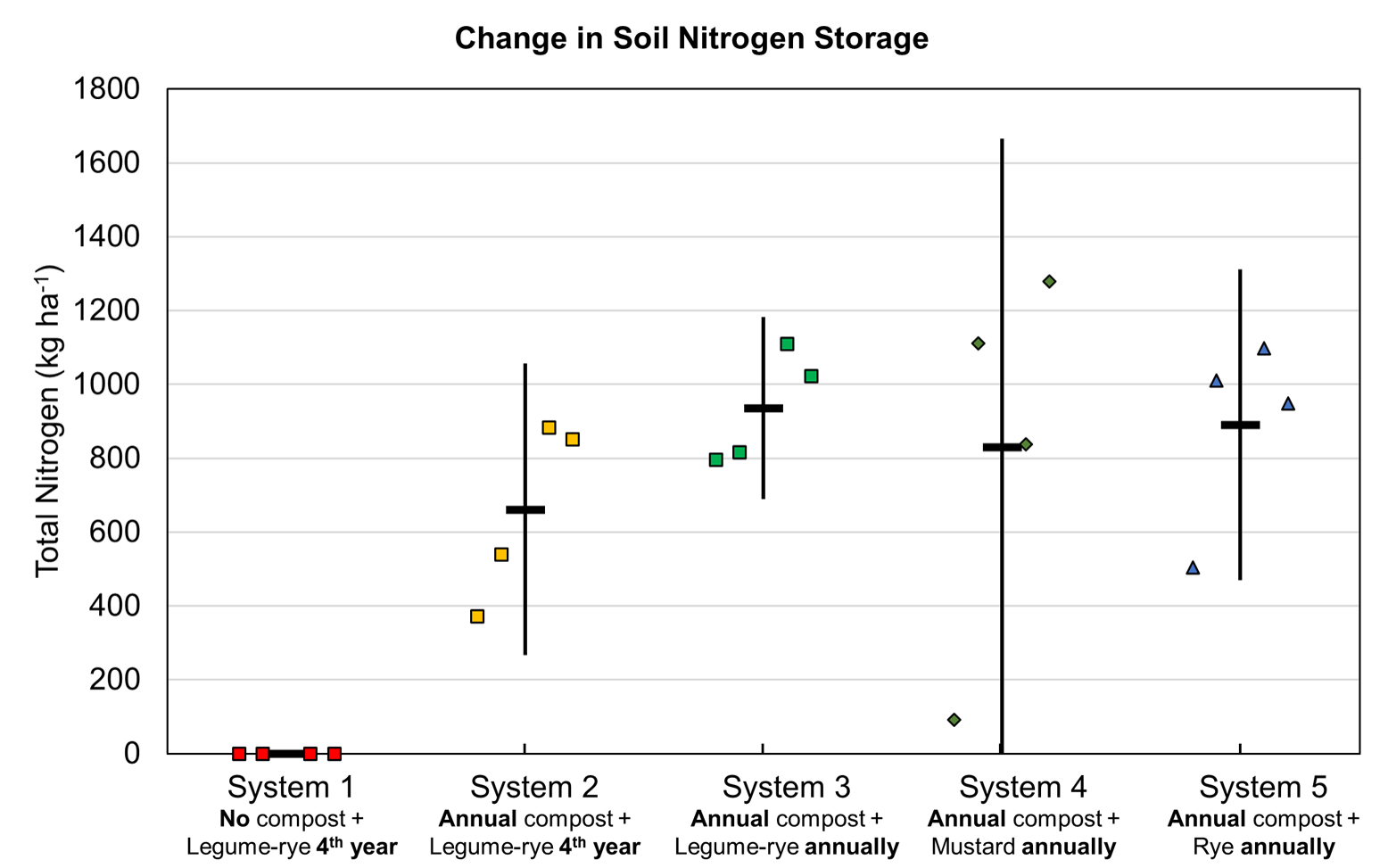

Supplement: S4 Fig — Mean soil nitrogen storage change between systems over eight years of vegetable production. Measurements were taken in five organic vegetable systems in Salinas, CA. Systems differed by annual compost additions (0 vs.7.6 Mg ha before each vegetable crop, oven-dry basis), cover crop type (legume-rye, mustard, or cereal rye alone) and cover cropping frequency (quadrennially vs. annually planted). System 1 = no compost+legume-rye 4th year; System 2 = annual compost+legume-rye 4th year; System 3 = annual compost+legume-rye annually; System 4 = annual compost+mustard annually; System 5 = annual compost+rye annually. Error bars are 95% confidence intervals (CI) with the mean at the horizontal line. Individual data points are averaged across years for replicates 1 through 4 of each system and are clustered around the mean in order from left to right so that they do no obscure the mean and CI. (TIF) [file pone.0267757.s004.tif]

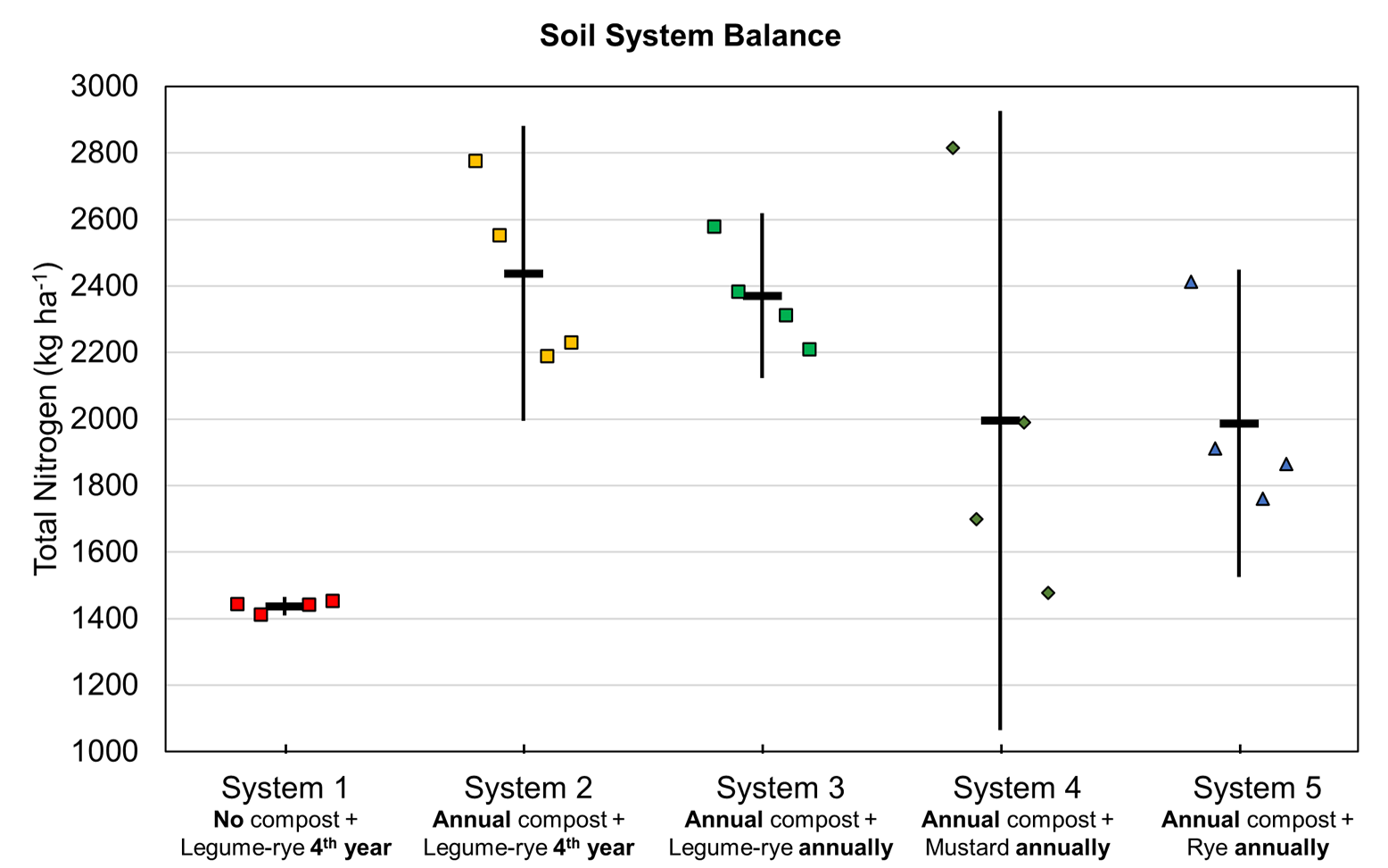

Supplement: S5 Fig — Measurements were taken in five organic vegetable systems in Salinas, CA. Systems differed by annual compost additions (0 vs.7.6 Mg ha before each vegetable crop, oven-dry basis), cover crop type (legume-rye, mustard, or cereal rye alone) and cover cropping frequency (quadrennially vs. annually planted). System 1 = no compost+legume-rye 4th year; System 2 = annual compost+legume-rye 4th year; System 3 = annual compost+legume-rye annually; System 4 = annual compost+mustard annually; System 5 = annual compost+rye annually. Error bars are 95% confidence intervals (CI) with the mean at the horizontal line. Individual data points are averaged across years for replicates 1 through 4 of each system and are clustered around the mean in order from left to right so that they do no obscure the mean and CI. (TIF) [file pone.0267757.s005.tif]
